# LncRNAs induce oxidative stress and spermatogenesis by regulating endoplasmic reticulum genes and pathways

**DOI:** 10.18632/aging.202971

**Published:** 2021-05-06

**Authors:** Tie-Cheng Sun, Yi Zhang, Kun Yu, Yao Li, Hong Yu, Shan-Jie Zhou, Ya-Peng Wang, Shou-Long Deng, Li Tian

**Affiliations:** 1 Reproductive Medical Center, Department of Obstetrics and Gynecology, Peking University International Hospital, Beijing 102206, China; 2 Reproductive Medicine Centre, Peking University Second Affiliated Hospital, Beijing 100044, China; 3 Institute of Laboratory Animal Sciences, Chinese Academy of Medical Sciences and Comparative Medicine Center, Peking Union Medical College, Beijing 100021, China; 4 Center of Reproductive Medicine, Peking University Third Hospital, Beijing 100191, China; 5 Department of Medicine, Panzhihua University, Sichuan 16700, China; 6 Beijing Key Laboratory for Animal Genetic Improvement, National Engineering Laboratory for Animal Breeding, Key Laboratory of Animal Genetics and Breeding of the Ministry of Agriculture, College of Animal Science and Technology, China Agricultural University, Beijing 100193, China

**Keywords:** long noncoding RNAs, oligozoospermia, spermatogenesis, oxidative stress, apoptosis

## Abstract

Oligozoospermia or low sperm count is a leading cause of male infertility worldwide. Despite decades of work on non-coding RNAs (ncRNAs) as regulators of spermatogenesis, fertilization, and male fertility, the literature on the function of long non-coding RNAs (lncRNAs) in human oligozoospermia is scarce. We integrated lncRNA and mRNA sequencing data from 12 human normozoospermic and oligozoospermic samples and comprehensively analyzed the function of differentially expressed lncRNAs (DE lncRNAs) and mRNAs (DE mRNAs) in male infertility. The target genes of DE lncRNAs were identified using a Gaussian graphical model. Gene ontology terms and Kyoto Encyclopedia of Genes and Genomes pathways were primarily enriched in protein transport and localization to the endoplasmic reticulum (ER). The lncRNA–mRNA co-expression network revealed cis- and trans-regulated target genes of lncRNAs. The transcriptome data implicated DE lncRNAs and DE mRNAs and their target genes in the accumulation of unfolded proteins in sperm ER, PERK-EIF2 pathway-induced ER stress, oxidative stress, and sperm cell apoptosis in individuals with oligozoospermia. These findings suggest that the identified lncRNAs and pathways could serve as effective therapeutic targets for male infertility.

## INTRODUCTION

**Figure 1:**
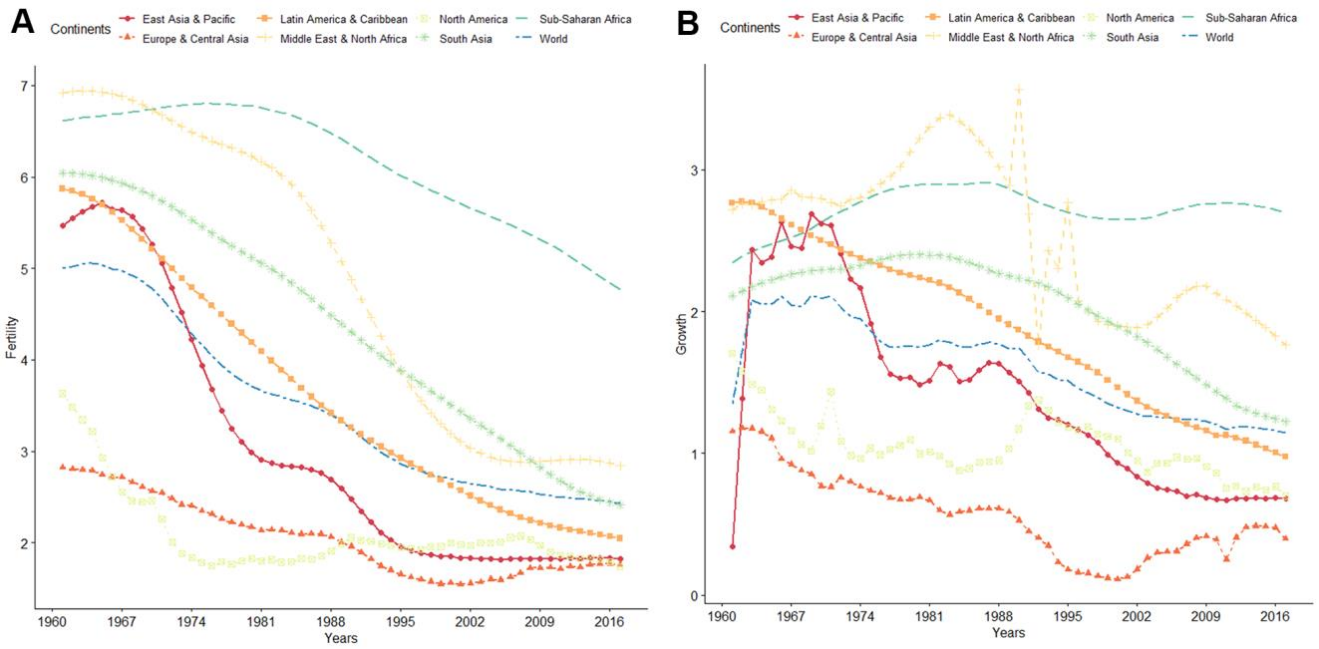
(**A**) WHO data on global trends of fertility from 1960 to 2016. (**B**) WHO data on global trends of population growth rate from 1960 to 2016.

Infertility is a global problem affecting human reproductive health. According to the World Health Organization, global fertility and population growth rates have continuously declined over the past 50 years (Figure 1). It is estimated that about 10 to 15% of couples in the reproductive ages are infertile, with approximately 40 to 50% of infertility cases attributed to “male factor” [1–4]. The most common causes of male infertility are low sperm concentration (oligozoospermia) and reduced sperm motility (asthenospermia), which often occur together [5, 6]. Oligozoospermia is characterized by qualitative and quantitative defects in spermatogenesis, manifested as poor sperm motility and morphology and sperm apoptosis [7]. Compared with healthy individuals, semen parameters, such as progressive rate, non-progressive rate, and DNA fragmentation index (DFI), are abnormal in those with oligozoospermia [7, 8], reducing the possibility of the sperm to reach the egg in the oviduct [6, 9–11]. Several studies have implicated chromosomal abnormalities, gene regulation disorders, environmental factors, infections, immune-related factors, and endocrine dysfunction in the etiology of oligozoospermia [6, 9].

Epigenetic changes are inherited modifications that regulate gene expression, without any alteration in the underlying nucleotide sequence [12–21]. Several external factors, such as diet, air pollution, and chemical exposure, influence epigenetic modifications [12, 17, 22]. Moreover, genetic studies report that various environmental factors affect gametogenesis, early sperm development, and sperm apoptosis by epigenetically reprogramming the genome and thus reducing the sperm count [12–20].

Non-coding RNAs (ncRNAs) regulate transcription and translation by inhibiting the binding of transcription factors to their target DNA. They can also epigenetically regulate gene expression by activating epigenetic modifiers to enhance DNA methylation and histone modifications [23–26]. Based on their length, ncRNAs are categorized as long ncRNAs (lncRNAs), short ncRNAs, and pseudogenes. Short ncRNAs such as miRNAs and PIWI-interacting RNAs (piRNAs) regulate the development of germ cells, primordial germ cell specialization, and early and late spermatogenesis, and are involved in the pathogenesis of various reproductive disorders [27–29]. LncRNAs—the largest category of ncRNAs—control the transcription factors linked to primordial germ cell specialization, such as BLIMP1, PRDM1, and DAZL, and maintain the survival of spermatogonial cells [27, 30–36].

High-throughput screening of bull semen transcriptome identified the function of ncRNAs in sperm motility [37]. We compared the sperm transcriptome profiles of patients with obstructive azoospermia (OA) (*n* = 3) and individuals with normal spermatogenesis (including the control group) to systematically define the expression profiles of lncRNAs and mRNAs. We next used these expression data to identify oligozoospermia-related lncRNAs and functional genes and their involvement in male infertility.

## RESULTS

### RNA sequencing and identification of DE mRNAs and DE lncRNAs

We performed a case–control study involving six normozoospermic (NS) samples from healthy fertile controls and six oligozoospermic (OS) samples from infertile patients to identify the major perturbations in oligozoosperms. Cases and controls were closely matched for age, body mass index (BMI), and sperm DFI (Table 1). The levels of reproductive hormones, such as follicle-stimulating hormone (FSH), luteinizing hormone (LH), and testosterone (T), were analyzed. As summarized in Table 1, we did not observe any differences in the levels of these hormones between NS and OS samples.

**Table 1:** Clinical data of participants.

**Parameters**	**Normospermia** **fertile controls (*n* = 6)**	**Oligozoospermia** **infertile group (*n* = 6)**	***p*-Value**
**Age (y)**	32.83 ± 4.96	37.67 ± 3.56	0.081
**BMI (kg/m^2^)**	24.32 ± 2.44	21.31 ± 2.68	0.069
**DFI (%)**	18.82 ± 14.58	35.54 ± 19.78	0.127
**FSH (IU mL^-1^)**	5.33 ± 2.17	9.11 ± 4.19	0.078
**LH (IU mL^-1^)**	4.11 ± 0.84	5.8 ± 4.3	0.366
**T (ng mL^-1^)**	4.0 ± 2.21	4.1 ± 2.34	0.943
**Volume (mL)**	4.22 ± 2.38	4.32 ± 2.01	0.939
**Concentration (Million mL^-1^)**	81.78 ± 37.21	8.83 ± 3.55	0.000
**PR (%)**	56.82 ± 17.16	32.01 ± 19.07	0.039
**NP (%)**	16.04 ± 6.05	8.6 ± 5.39	0.048
**Motility (%)**	72.86 ± 16.59	40.62 ± 23.31	0.020
**Normal morphology (%)**	4.83 ± 0.52	3.93 ± 0.71	0.031

Abbreviations: BMI, body mass index; DFI, DNA fragmentation index; FSH, follicle-stimulating hormone; LH, luteinizing hormone; PR, progressive rate; NP, non-progressive rate; T, testosterone.

We next assessed the association between semen characteristics and oligozoospermia. Five of the six examined semen characteristics (concentration, progressive rate, non-progressive rate, motility, and normal morphology) differed between the two groups (*p* = 0.0001–0.048; Table 1). Compared with individuals in the OS group, those in the NS group had a 9-fold higher concentration of collected sperms (NS: 81.78 ± 37.21; OS: 8.83 ± 3.55). Similar patterns were observed for other characteristics (progressive rate, non-progressive rate, motility, and normal morphology) using the *t*-test (Table 1). The remaining characteristic, ejaculate volume, was not associated with the development of oligozoospermia (*p* = 0.939).

**Figure 2:**
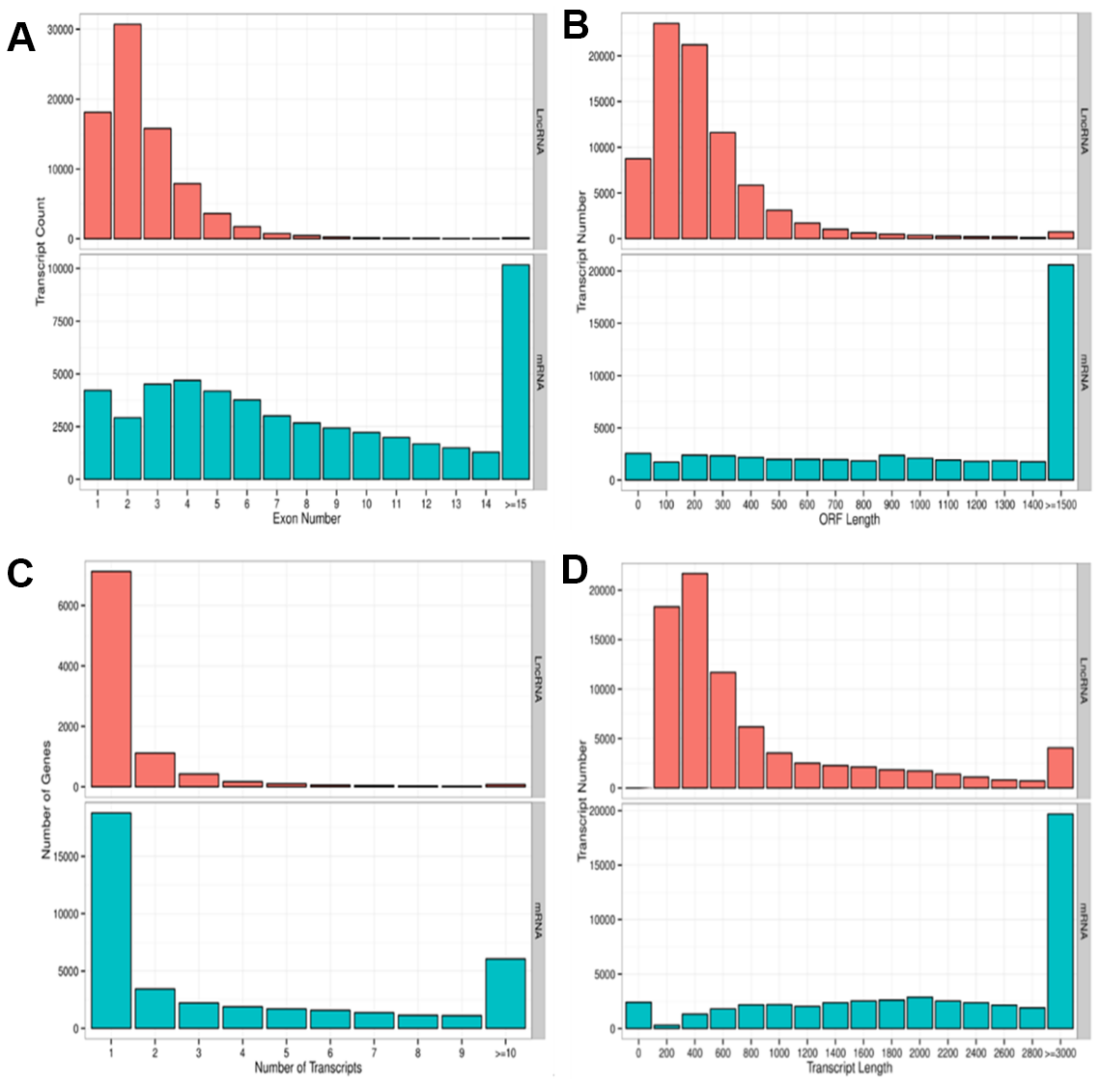
**Parameters of lncRNA and mRNA transcripts.** (**A**) Distribution pattern of exon numbers of lncRNA and mRNA transcripts. (**B**) Distribution pattern of ORF length of lncRNA and mRNA transcripts. (**C**) Distribution pattern of gene numbers of lncRNA and mRNA transcripts. (**D**) Distribution pattern of transcript lengths of lncRNA and mRNA transcripts.

We performed a high-throughput whole transcriptome shotgun sequencing to compare the sperm RNA expression patterns between NS and OS groups. We extracted high-quality RNA from each sperm sample and constructed cDNA libraries. Next, we calculated the levels of transcripts of lncRNAs and mRNAs on an Illumina HiSeq X-Ten Platform, which supported a read length of 2 × 150 bp with quality scores of ≥ 75% of bases above Q30. After filtering the raw data, 24.4 to 79.8 million clear reads and 2,501 lncRNAs and mRNAs were identified distribution of base composition and distribution of quality are shown in Figure 2. Figure 2 shows the characteristics of identified lncRNAs and mRNAs. The lncRNA transcripts were shorter in length (Figure 2D) and contained fewer exons than mRNAs (Figure 2A). The majority of identified lncRNAs contained one to two transcripts, whereas most mRNAs had one to four transcripts (Figure 2C)—findings that are in agreement with those of previous studies [30–32]. The length of open reading frames (ORFs) of mRNAs was shorter than that of lncRNA ORFs (Figure 2B).

### LncRNA and mRNA characterization

**Figure 3:**
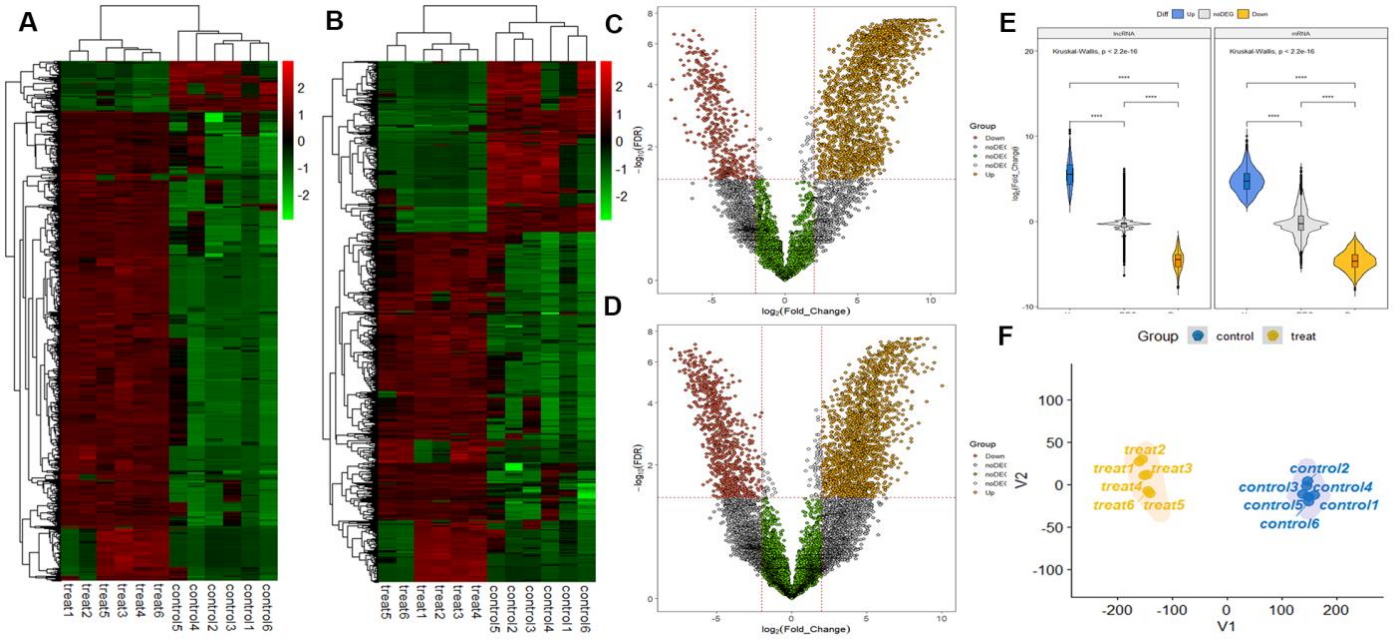
**LncRNA and mRNA profiles based on RNA sequencing data.** (**A**) Comparison of two-dimensional hierarchical clustering of distinguishable lncRNA expression profiles in individuals with oligozoospermia and controls. Red indicates high expression, green indicates low expression. Probes are shown in rows, and samples are shown in columns. (**B**) Two-dimensional hierarchical clustering of distinguishable mRNA expression profiles. (**C**) Volcano plot of differentially expressed lncRNAs in individuals with oligozoospermia compared with normal controls. Red points represent downregulated lncRNAs and yellow points represent upregulated lncRNAs in individuals with oligozoospermia with a greater than 2.0-fold change. (**D**) Volcano plot of differentially expressed mRNAs. (**E**) Violin plot of lncRNA and mRNA profiles in individuals with oligozoospermia. (**F**) t-distributed stochastic neighbor embedding (t-SNE) plot of samples based on lncRNA and mRNA profiles in individuals with oligozoospermia.

**Figure 4:**
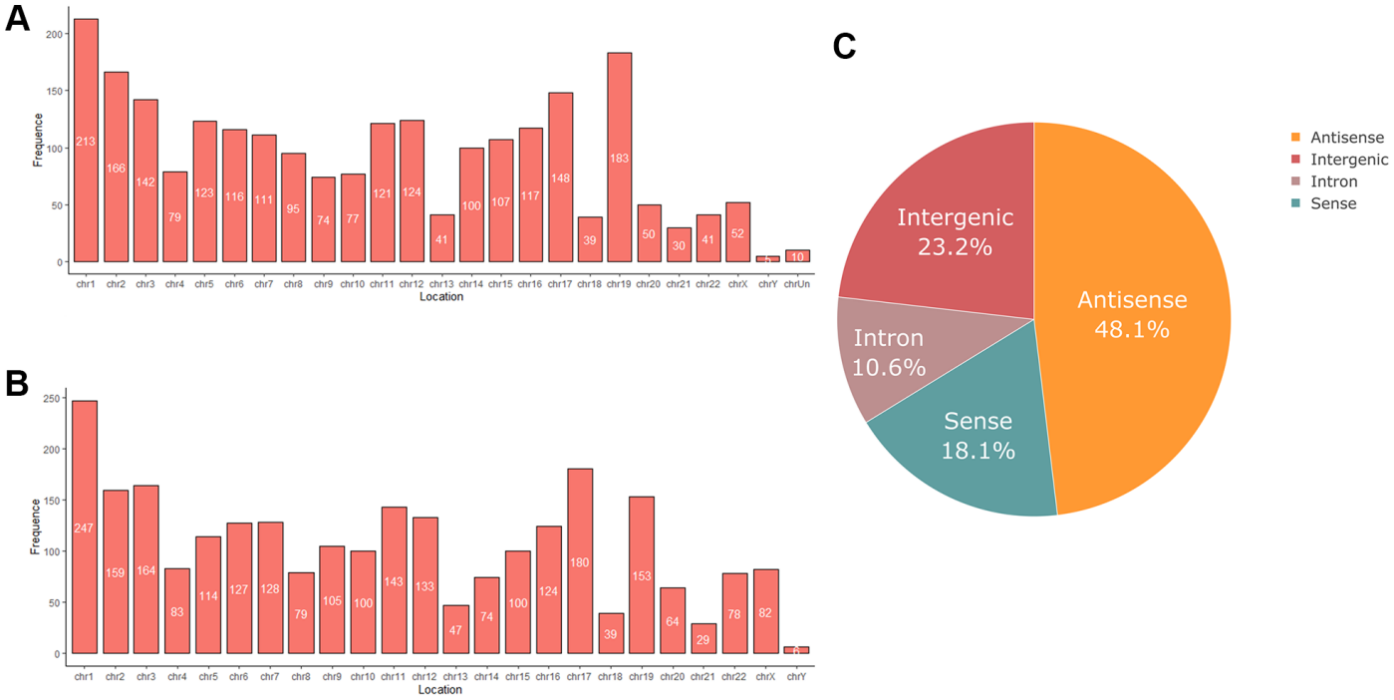
(**A**) Chromosome distribution of differentially expressed lncRNAs in oligozoospermia. (**B**) Chromosome distribution of differentially expressed mRNAs in oligozoospermia. (**C**) Pie chart of differentially expressed lncRNAs identified in different subgroup categories.

RNA sequencing analysis revealed that of the 55,278 identified lncRNAs and mRNAs, 4,963 were differentially expressed (DE) in OS samples as compared with NS samples (2,364 lncRNAs and 2,599 mRNAs; Supplementary Tables 1, 2). Of these, only 464 lncRNAs and 875 mRNAs were downregulated; 1,900 lncRNAs and 1,724 mRNAs were upregulated (Supplementary Tables 1, 2). Moreover, 772 lncRNAs and 410 mRNAs were identified in oligozoosperms with a log2 fold change (FC) > 6 or < –6 compared with normozoosperms (Supplementary Table 2 and Figure 3). The most upregulated lncRNA was lnc-GNS-3 (FC = 10.0921, FDR = 2.91E-08) and the most downregulated lncRNA was ITGA9-AS1 (FC = –6.2743, FDR = 1.60E-07). The most upregulated mRNA was ZNF350 (FC = 7.3363, FDR = 3.16E-08) and the most downregulated mRNA was ASPH (FC = –6.4339, FDR = 7.92E-08). The DE lncRNAs and DE mRNAs were widely scattered among all chromosomes although the distribution was unequal (Supplementary Tables 3, 4). Chromosome 1 had the highest number of altered RNAs, 213 lncRNAs and 247 mRNAs that accounted for 9.01% (213/2,364) and 9.50% (247/2,599) of all DE lncRNAs and DE mRNAs, respectively (Figure 4A, 4B). The X chromosome had the highest number of DE lncRNAs and DE mRNAs (52 and 82) than the Y chromosome (5 and 6) (Figure 4A, 4B). Box plots revealed the lncRNA and mRNA expression patterns in each sperm sample (Figure 2E). Furthermore, analysis using PhastCons software revealed that lncRNA transcripts were less conserved than mRNA transcripts (Figure 2F).

### Classification of DE lncRNAs and DE mRNAs

We next classified DE lncRNAs and DE mRNAs and performed subgroup analysis to study their potential functions (Tables 2, 3). The DE lncRNAs were distributed into four subgroups: sense, antisense, intron, and intergenic (Supplementary Tables 5, 6). Antisense lncRNAs accounted for 48.1% of the total lncRNAs (1,077/1,900 upregulated and 60/464 downregulated), whereas intergenic lncRNAs constituted the second largest category (315/1,900 upregulated and 233/464 downregulated) (Figure 4C). Approximately 10.6% of the lncRNAs belonged to the intron subgroup (199/1,900 upregulated and 51/464 downregulated) (Figure 4C) and 18.1% lncRNAs were identified with sense regulation (309/1,900 upregulated and 120/464 downregulated) (Figure 4C).

**Table 2:** Top differentially expressed lncRNAs in oligozoospermia.

**LncRNA ID**	**Regulation**	**Fold change**	***p*-Value**	**Corr P**	**Type**
lnc-GNS-3	Upregulation	10.09212	1.44E-12	2.91E-08	Intergenic
XLOC_515910	Upregulation	8.866827	4.46E-12	2.91E-08	Sense
lnc-SERHL2-7	Upregulation	10.29479	5.34E-12	2.91E-08	Antisense
lnc-IL31RA-1	Upregulation	9.21277	6.47E-12	2.91E-08	Intron
lnc-NLRP2-2	Upregulation	7.527131	7.31E-12	2.91E-08	Antisense
lnc-CDK12-2	Upregulation	9.47426	7.56E-12	2.91E-08	Antisense
XLOC_1093926	Upregulation	7.485795	7.96E-12	2.91E-08	Intron
lnc-LRRC38-2	Upregulation	8.591819	8.33E-12	2.91E-08	Antisense
lnc-FOXN1-1	Upregulation	8.90089	8.60E-12	2.91E-08	Antisense
lnc-SLC46A2-1	Upregulation	9.376134	8.89E-12	2.91E-08	Antisense
lnc-CSNK1A1-7	Upregulation	8.601429	9.60E-12	2.91E-08	Antisense
lnc-PHLDB1-1	Upregulation	8.974802	9.65E-12	2.91E-08	Antisense
lnc-KB-1980E6.3.1-6	Upregulation	7.757378	1.04E-11	2.91E-08	Antisense
lnc-LAT2-2	Upregulation	7.96087	1.04E-11	2.91E-08	Intron
XLOC_2394941	Upregulation	8.509539	1.24E-11	3.12E-08	Intergenic
lnc-EEF1B2-3	Upregulation	7.892956	1.30E-11	3.12E-08	Antisense
lnc-AP3S1-15	Upregulation	9.267483	1.34E-11	3.12E-08	Antisense
lnc-SLA2-1	Upregulation	6.812729	1.68E-11	3.31E-08	Intron
lnc-RAPH1-6	Upregulation	6.822993	1.76E-11	3.31E-08	Antisense
lnc-TICAM1-1	Upregulation	8.925062	1.77E-11	3.31E-08	Antisense

**Table 3:** Top differentially expressed mRNAs in oligozoospermia.

**Gene ID**	**Gene symbol**	**Regulation**	**Fold change**	***p*-Value**	**Corr P**
59348	ZNF350	Upregulation	7.336254	1.43E-11	3.16E-08
283651	HMGN2P46	Upregulation	9.026764	3.58E-11	3.36E-08
146956	EME1	Upregulation	7.822658	3.68E-11	3.36E-08
164633	CABP7	Upregulation	8.243164	3.69E-11	3.36E-08
125111	GJD3	Upregulation	8.452515	3.77E-11	3.36E-08
692094	MSMP	Upregulation	8.397109	4.12E-11	3.45E-08
8896	BUD31	Upregulation	6.498333	8.85E-11	5.68E-08
148808	MFSD4	Upregulation	7.073111	8.85E-11	5.68E-08
11267	SNF8	Upregulation	5.932717	9.18E-11	5.68E-08
51003	MED31	Upregulation	6.986587	9.65E-11	5.68E-08
3206	HOXA10	Upregulation	7.704079	1.03E-10	5.68E-08
51399	TRAPPC4	Upregulation	7.058648	1.43E-10	6.68E-08
100130771	EFCAB10	Upregulation	7.244548	1.48E-10	6.78E-08
340719	NANOS1	Upregulation	7.507104	1.50E-10	6.78E-08
51282	SCAND1	Upregulation	6.752215	1.54E-10	6.86E-08
29893	PSMC3IP	Upregulation	8.194087	1.81E-10	7.76E-08
444	ASPH	Downregulation	–6.434	1.89E-10	7.92E-08
529	ATP6V1E1	Upregulation	6.463281	1.96E-10	8.02E-08
6202	RPS8	Upregulation	8.205232	1.98E-10	8.04E-08
220988	HNRNPA3	Upregulation	8.786221	2.13E-10	8.43E-08

### Enrichment analysis of DE mRNAs and DE lncRNAs

**Figure 5:**
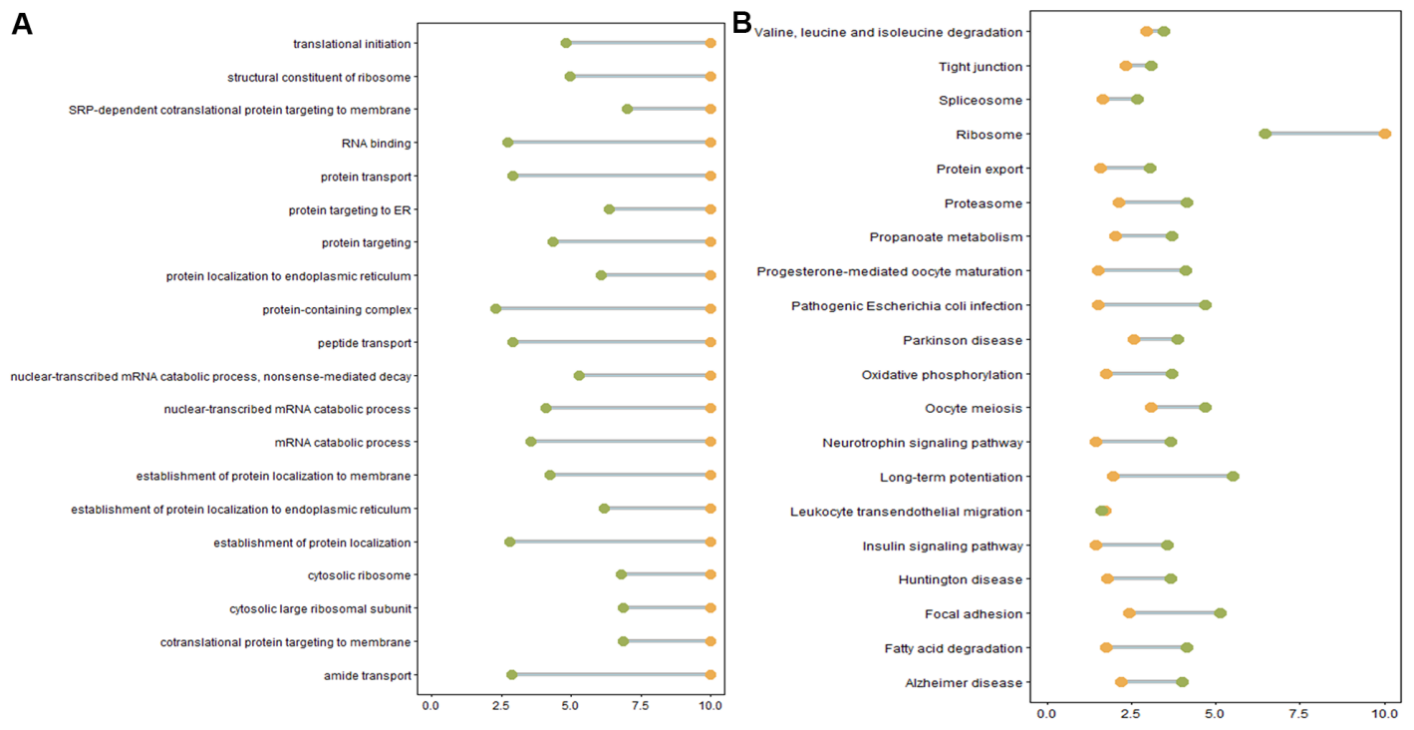
(**A**) Enriched GO terms. Green: Log10 *p*-value from over-representation analysis (ora), orange: Log10 *p*-value from pathway-level analysis (plage). (**B**) Enriched KEGG pathways. Green: Log10 *p*-value from over representation analysis (ora), orange: Log10 *p*-value from pathway-level analysis (plage).

We next performed GO and KEGG pathway enrichment analyses to study the functions of DE genes corresponding to DE mRNAs and DE lncRNAs. Over-representation (ora) GO analysis revealed 777, 196, and 188 terms enriched in biological processes (BP), cell components (CC), and molecular functions (MF), respectively. Similar results were obtained in the pathway-level analysis (plage). These GO terms were primarily related to intracellular protein transport and localization. GO terms with the highest differential expression were enriched in SRP-dependent co-translational protein targeting to the membrane, co-translational protein targeting to the membrane, and protein targeting to the ER (Figure 5A). Table 4 lists the DE genes associated with these processes, including CHMP4B, SEC61A1, SEC61A2, SEC61G, SEC62, SGTB, SPCS1, SRP9, SRP19, RYR2, ANK2, DDRGK1, INSIG1, KDELR2, RER1, and RTN4. Furthermore, differential GO terms were enriched in ER stress, oxidative stress, protein unfolding, and cell apoptosis (Figure 5B, Supplementary Table 7). Twenty-six over-represented GO terms were directly related to negative regulation of response to ER stress (Supplementary Table 7). In the case of protein unfolding, unfold protein binding had a significant difference (Supplementary Table 7). Five GO terms were related to oxidative stress, including neuron death in response to aerobic stress, negative regulation of aerobic stress-induced intrinsic acoustic signaling pathway, positive regulation of aerobic-stress induced intrinsic acoustic pathway, and regulation of oxidative stress-induced neuron death (Supplementary Table 7). Other apoptosis GO terms were those involved in the negative regulation of apoptosis (Supplementary Table 7).

**Table 4:** Expression profile of crucial genes involved in protein transport and localization.

**Gene ID**	**Gene symbol**	**Regulation**	**Fold change**	***p*-Value**	**Corr P**
128866	CHMP4B	Upregulation	4.641415	1.26E-08	9.73E-07
29927	SEC61A1	Upregulation	4.333018	2.73E-06	7.21E-05
55176	SEC61A2	Upregulation	7.408156	3.36E-05	0.000613
23480	SEC61G	Upregulation	2.779526	0.009469	0.070513
7095	SEC62	Upregulation	4.989299	0.000227	0.003211
6449	SGTA	None	−1.45366	0.079382	0.318414
54557	SGTB	Upregulation	2.447903	0.008083	0.062471
28972	SPCS1	Upregulation	4.387165	5.97E-05	0.001012
9789	SPCS2	None	−1.45238	0.443984	0.607944
60559	SPCS3	None	3.084166	0.071509	0.301378
6726	SRP9	Upregulation	6.771347	1.96E-05	0.000387
6727	SRP14	None	3.593638	0.05458	0.251185
6728	SRP19	Upregulation	3.648171	0.006099	0.04998
6731	SRP72	None	3.660797	0.05925	0.266326
6729	SRP54	None	0.969371	0.671303	0.769497
6747	SSR3	None	3.791997	0.082548	0.320391
23471	TRAM1	None	2.082365	0.305616	0.487579
9697	TRAM2	None	−0.83275	0.089043	0.320391
6262	RYR2	Downregulation	−5.41927	0.000268	0.003698
287	ANK2	Downregulation	−7.4485	3.98E-09	4.25E-07
65992	DDRGK1	Downregulation	−3.63455	0.002235	0.022008
3638	INSIG1	Upregulation	4.908842	0.004901	0.04179
11014	KDELR2	Upregulation	3.895493	0.004696	0.040402
11079	RER1	Upregulation	4.332667	0.001388	0.014811
57142	RTN4	Upregulation	3.391149	0.021874	0.133722
202018	TAPT1	None	−2.50812	0.086066	0.320391
337867	UBAC2	None	2.42096	0.081385	0.320391

Differentially expressed genes involved in these processes are listed in Tables 5, 6. These included upregulated EIF2a, ATF5, ATF4, XBP1, SERP1, and CEBPZ associated with ER stress. Only a few genes, such as MAPK8, were downregulated (Table 5). Although EIF2AK3, an ER stress-related gene, was not differentially expressed (Table 5), its homologous gene EIF2AK1 was upregulated (Table 5). Similarly, although ERN1 was not differentially expressed, it displayed more than 2-fold change (Table 5). Oxidative stress-related DE genes included SOD1, SOD2, NOXA1, XDH, and MAPK14 (Table 6). The expression of SOD1, SOD2, MAPK14, NFKB1, and SP1 was upregulated (Table 6), whereas that of NOX1, XDH, NFE2L1, and NCF1C was downregulated (Table 6). HOMX2 was upregulated but not HMOX1 (Table 6). Similarly, GPX3 was not differentially expressed; however, GPX1 and GPX4 were upregulated (Table 6). Further, certain apoptotic genes, such as CASP6, MCL1, NFKB1, BIRC2, AKT3, GADD45G, CAPN1, EIF2A, RAF1, MAP2K1, ENDO, and TNFRSF1A, were differentially expressed (Supplementary Table 2).

**Table 5:** Expression profile of crucial genes involved in endoplasmic reticulum stress.

**Gene ID**	**Gene symbol**	**Regulation**	**Fold change**	***p*-Value**	**Corr P**
83939	EIF2A	Upregulation	7.368758342	1.46E-09	2.27E-07
22809	ATF5	Upregulation	6.571856	7.47E-08	3.74E-06
468	ATF4	Upregulation	3.978785	0.034773	0.19102
7494	XBP1	Upregulation	5.115628	1.66E-05	0.000336
27230	SERP1	Upregulation	7.627359469	2.28E-05	0.000439433
487	ATP2A1	Upregulation	2.138295777	2.31E-05	0.000443061
5599	MAPK8	Downregulation	−4.53268	0.001238	0.013433
10153	CEBPZ	Upregulation	9.508285	1.42E-08	1.08E-06
10018	BCL2L11	Upregulation	4.309704	0.000362	0.004786
840	CASP7	Upregulation	4.335948	0.009178	0.069068
4170	MCL1	Upregulation	5.38951	0.003147	0.029303
2081	ERN1	None	−2.00713	0.075075	0.309882
27102	EIF2AK1	Upregulation	5.956155	0.000597	0.007317
9451	EIF2AK3	None	−0.95555	0.134954	0.373771

**Table 6:** Expression profile of crucial genes involved in oxidative stress.

**Gene ID**	**Gene symbol**	**Regulation**	**Fold change**	***p*-Value**	**Corr P**
6647	SOD1	Upregulation	8.027872	1.13E-06	3.44E-05
6648	SOD2	Upregulation	4.926199	9.72E-05	0.001556
6649	SOD3	None	0.094519	0.925824	0.944478
27035	NOX1	None	−0.53031	0.716694	0.809006
79400	NOX5	None	0.072599	0.957191	0.970732
10811	NOXA1	Downregulation	−1.71036	0.04837	0.23708
7498	XDH	Downregulation	−4.39865	0.001058	0.011869
1432	MAPK14	Upregulation	4.671004	2.05E-05	0.000401
4779	NFE2L1	Downregulation	−5.56491	2.03E-06	5.60E-05
4780	NFE2L2	None	0.571726	0.750761	0.834931
4790	NFKB1	Upregulation	3.770259	3.26E-05	0.000598
6667	SP1	Upregulation	6.560468	7.33E-07	2.41E-05
25828	TXN2	Upregulation	4.647904	1.06E-07	5.00E-06
2729	GCLC	Upregulation	4.867694	1.73E-07	7.47E-06
3163	HMOX2	Upregulation	4.786777	1.21E-08	9.50E-07
3162	HMOX1	None	−0.12542	0.655116	0.769497
2879	GPX4	Upregulation	5.109184	1.16E-05	0.000246
2876	GPX1	Upregulation	3.138132	8.36E-05	0.001362
2878	GPX3	None	1.266242	0.397058	0.566021
2936	GSR	Upregulation	5.151825	0.007639	0.05978
654817	NCF1C	Downregulation	−3.02159	0.001373	0.014687
653361	NCF1	None	0.043399	0.896427	0.919911
4688	NCF2	Downregulation	−3.89704	0.005773	0.047846
4784	NFIX	Upregulation	4.261149	0.00448	0.03892
2305	FOXM1	Downregulation	−4.02428	0.013965	0.09426

The majority of DE genes were also enriched in the identified KEGG pathways, such as Alzheimer’s disease pathway, Parkinson’s disease pathway, ribosome and fatty acid degradation pathway (Supplementary Table 8). Alzheimer’s disease and Parkinson’s disease pathways were associated with ER stress or oxidative stress-induced cell apoptosis, consistent with the previous GO terms analysis results (Supplementary Table 7). However, certain genes involved in ER stress and oxidative stress initiation in these pathways, such as APP and PRKN, were not differentially expressed in oligozoospermic individuals (Supplementary Table 2).

### Co-expression analysis of DE lncRNAs

**Figure 6:**
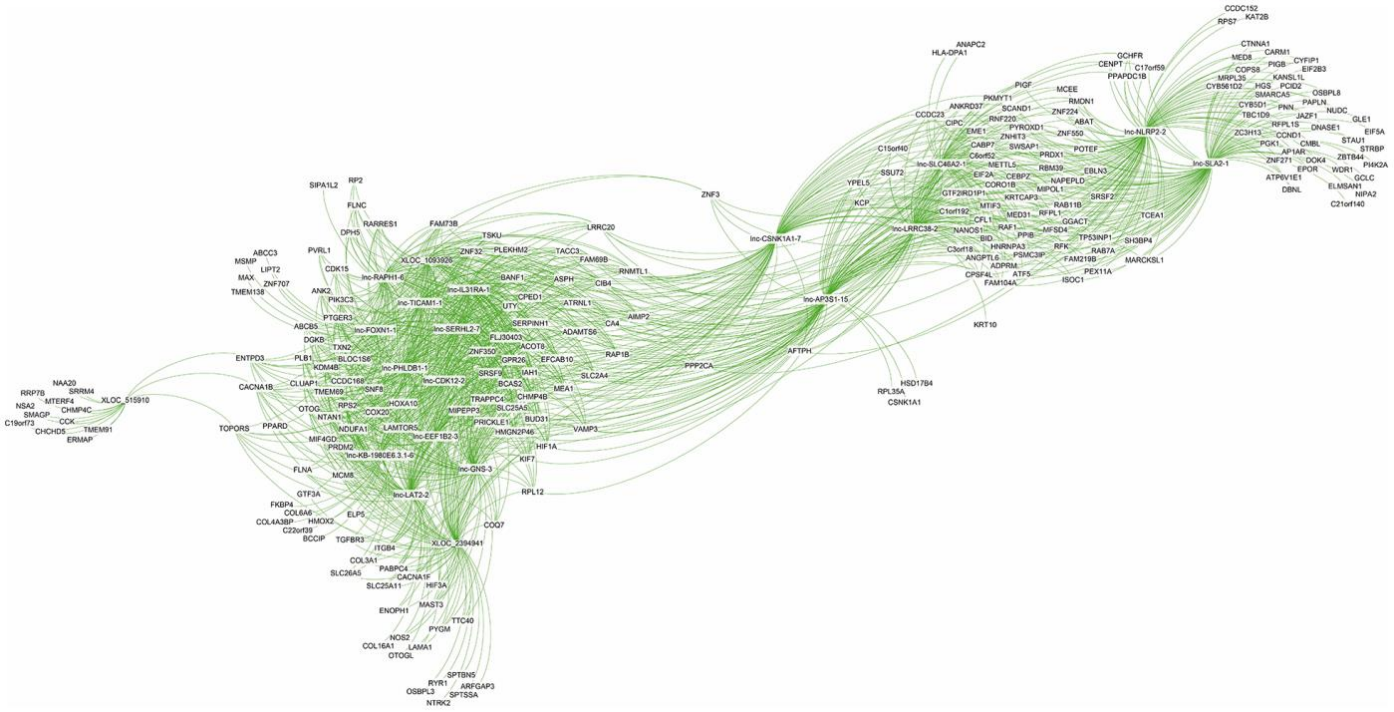
Co-expression subnetwork of the top 20 differentially expressed lncRNAs.

The co-expression network analysis of lncRNAs and mRNAs in oligozoospermic individuals revealed potential internal adjustment mechanisms. Several lncRNAs correlated with a single mRNA and vice versa. For example, 21,468 lncRNA and mRNA partial correlation pairs were found for the top 20 DE lncRNAs (Supplementary Table 9). Of these, 1,542 pairs showed a strong correlation coefficient, 8,421 pairs showed a moderate correlation coefficient, and 11,505 pairs had a weak correlation coefficient (Supplementary Table 9). The majority of these lncRNAs had common correlated genes. For example, the upregulated lncRNA lnc-GNS-3 correlated with target genes BANF1, TXN2, PIK3C3, and KDM4b, which also strongly correlated with other upregulated lncRNAs, namely lnc-SERHL2-7, lnc-PHLDB1-1, and lnc-IL31RA-1 (Figure 6 and Supplementary Table 9). However, the majority of genes associated with XLOC_515910 and lnc-NLRP2-2, such as CHCHD5 and KAT2B, only correlated with one of the top DE lncRNAs (Figure 6, Supplementary Table 9).

LncRNAs strongly correlating with EIF2A, a major ER stress gene, were lnc-FAM198B-1, lnc-C4orf21-1, lnc-SPIN3-5, and lnc-AC006050.2.1-6 (Supplementary Tables 10, 11, 22). Those with a moderate negative correlation included lnc-TMEM75-8, lnc-CYB5R2-3, XLOC_2993599, and lnc-MPP7-8 (Table 7). LncRNAs with moderate positive correlation included lnc-TOR3A-1, lnc-STARD9-1, lnc-SLC25A47-5, and XLOC_002818 (Table 7). Lnc-TULP4-1, lnc-PTPN2-6, FOXP1-AS1, and lnc-ZNF101-4 showed a strong positive correlation with oxidative stress-related gene SOD1 (Table 7). LncRNAs with moderate negative correlation with SOD1 included XLOC_1174819, XLOC_2396188, XLOC_2837388, and XLOC_23762 (Table 7). Those with moderate positive correlation included lnc-BCAP31-1, lnc-C2orf84-1, XLOC_2394463, and lnc-ADAM21-4. SRP9, a gene involved in protein localization, positively correlated with lnc-IARS2-2, lnc-WDR77-2, lnc-C1QL3-1, and lnc-SLC36A1-5 (Table 7). It showed a moderate negative correlation with lnc-PPP1R3G-3, lnc-GGCT-1, XLOC_2028132, and lnc-MPP7-8 (Table 7). Lnc-RAD9B-1, lnc-ZNF687-1, lnc-SLC3A2-3, and lnc-MAP6-1 exhibited a moderate positive correlation with SRP9. ER stress, oxidative stress, and protein localization shared several lncRNAs (Table 7). For example, ADIRF-AS1 not only controlled EIF2A and EIF2AK1 in ER stress but was also related to NCF2 and NCF1C of oxidative stress, and SPCS1 and UBAC2 of protein transport (Supplementary Tables 12, 13, and Table 7).

**Table 7:** Expression profile of crucial genes involved in spermatogenesis.

**Gene ID**	**Gene symbol**	**Regulation**	**Fold change**	***p*-Value**	**Corr P**
3207	HOXA11	Upregulation	2.630147	0.006865	0.055022
3235	HOXD9	Downregulation	–2.95139	0.034474	0.18968
3973	LHCGR	None	–2.01129	0.0785	0.317254
4214	MAP3K1	Downregulation	–3.50326	0.001029	0.011594
6666	SOX12	Downregulation	–2.88106	0.011734	0.082572
6660	SOX5	Downregulation	–2.5398	0.077814	0.316286
64321	SOX17	Downregulation	–2.39059	0.037155	0.200292
374955	SPATA21	Downregulation	–6.26095	1.07E-06	3.32E-05
65244	SPATS2	Downregulation	–4.60831	0.00102163	0.011516328
402381	SOHLH1	Downregulation	–3.55715	0.066831	0.289663
117	ADCYAP1R1	Downregulation	–3.10056	0.010437	0.075885
7301	TYRO3	Downregulation	–3.14978	0.00333	0.030655
558	AXL	Upregulation	2.947083	5.41E-05	0.000929
6677	SPAM1	Downregulation	–2.01802	0.001119	0.012398
57504	MTA3	Downregulation	–4.65991	1.65E-07	7.20E-06

To study the function of lncRNAs in gene regulation and pathogenesis of oligozoospermia, cis- and trans- predictions were performed based on co-expression network analysis. All top 20 DE lncRNAs were predicted to have trans-regulated target genes. Lnc-TICAM1-1 and lnc-PHLDB1-regulated the expression of KDM4B and TRAPPC4 with cis-interaction effect (Supplementary Table 14). Further, lnc-TICAM1-1 may be regulated by transcription factors LF-A1, p300, and EllaE-A (Supplementary Table 14).

**Figure 7:**
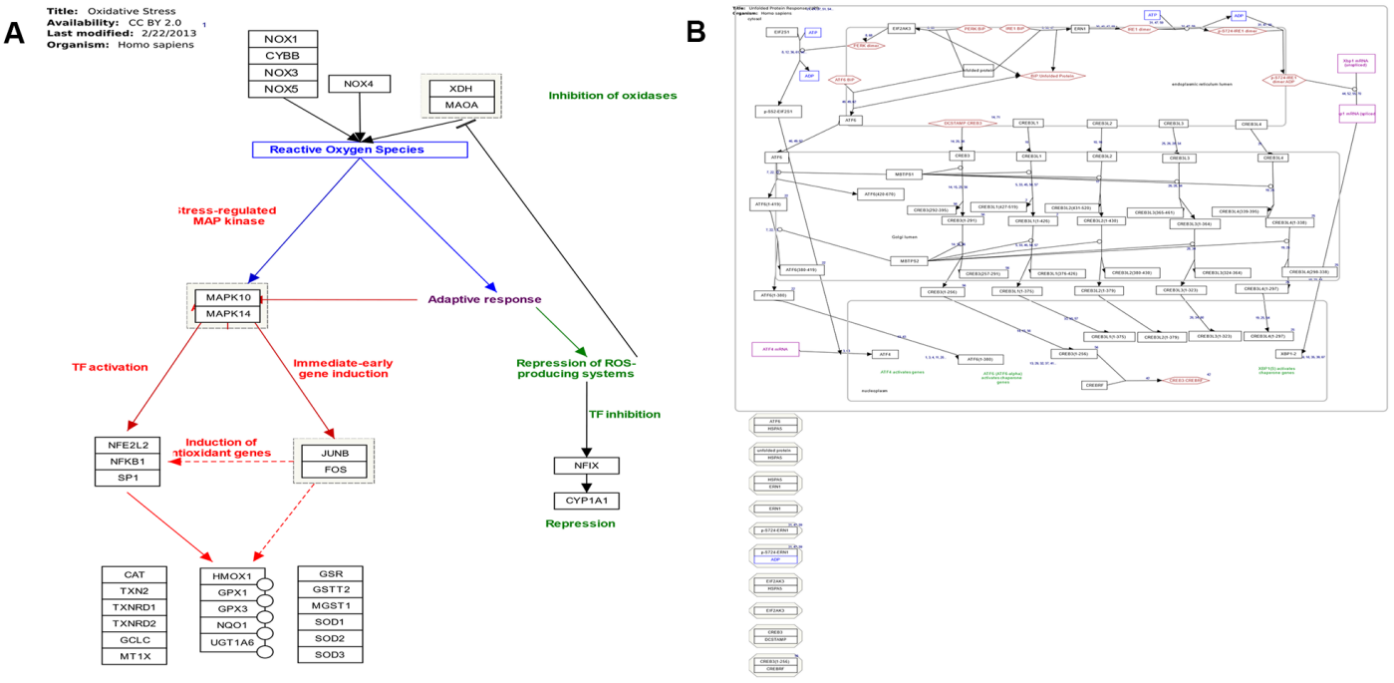
(**A**) Pathway map of oxidative stress (adapted from Wikipathway database). (**B**) Pathway map of endoplasmic reticulum stress and unfolded protein response (adapted from Wikipathway database).

The majority of identified lncRNAs in ER stress, oxidative stress, and protein transport exerted trans-regulation effects on target genes; however, no lncRNA displayed cis-regulation effect (Supplementary Tables 15–18, 22, and Figure 7A). LncRNAs with trans-regulation effect included lnc-TSKS-1, lnc-PRR15L-2, and lnc-NT5DC2-1 (Supplementary Tables 15–19). The ER stress-related lnc-TSKS-1 may be regulated by ELLaE-A, E47, Elk-1, and other transcription factors (Supplementary Table 20 and Figure 7B). The oxidative stress-related lnc-PRR15L-2 may be correlated with transcription factors C/EBPalpha, C/EBPbeta, and wt1. L (Supplementary Table 20). Other transcription factors such as p300, c-Ets-1, and R2 may regulate lnc-NT5DC2-1 involved in protein transport (Supplementary Table 21).

**Figure 8:**
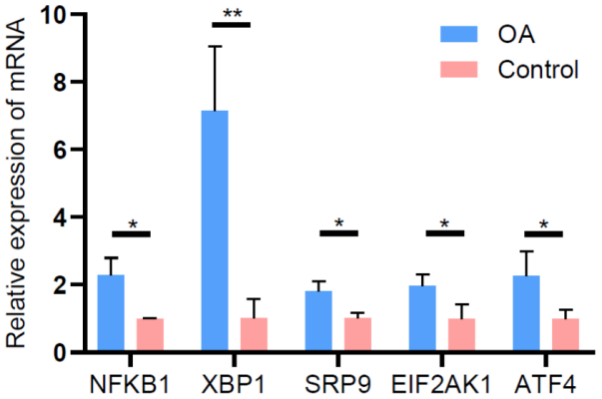
Validation of differentially expressed lncRNAs by qRT-PCR.

To validate the reliability of the lncRNA microarray data, we selected five upregulated lncRNAs (NFKB1, XBP1, SRP9, EIF2AK1, and ATF4) that were abundantly and differentially expressed (FCs > 6.0). qRT-PCR was used to analyze the differences in their expression. The results of the qRT-PCR analysis were largely consistent with those of the microarray data (Figure 8).

## DISCUSSION

Non-coding RNAs participate in the pathogenesis and development of several reproductive diseases. For example, certain miRNAs, such as mir-290-295 and mir-34c, are involved in spermatogenesis and oogenesis [27, 38, 39], and their dysregulation results in primordial germ cell migration disorders or downregulated expression of Nanos2 in spermatogonial stem cells [27, 38, 39]. We first screened the whole genome expression patterns of lncRNAs and mRNAs in spermatozoa of patients with oligospermia and normal individuals. We next compared the expression profiles of lncRNAs and mRNAs between the two groups using bioinformatics and systematically analyzed the characteristics of oligozoospermia-related lncRNAs and mRNAs [40, 41].

Our results revealed that 2,364 lncRNAs and 2,599 mRNAs were abnormally expressed in patients with oligozoospermia. The proportion of downregulated lncRNAs and mRNAs was less than that of upregulated ones. Furthermore, we did not find any difference in the expression of lncRNAs by qRT-PCR and RNA-sequencing [40, 41], which could be attributed to different normalization processes used in the two methods [41–43].

The chromosomal distribution of DE lncRNAs and DE mRNAs was uneven, with the majority of them located on chromosomes 1, 17, and 19. The number of differential genes on the X chromosome was higher than that on the Y chromosome [44]. Bache conducted a cohort study of male infertility and reported 5 out of 10 disease-related autosomal bands on chromosome 1 [45], suggesting that chromosome 1 contains a domain regulating the male reproductive capacity. Further, Bache reported that men with pericentric inversion of chromosome 1 were at the risk of developing infertility due to spermatogenesis dysfunction [46]. Similar findings were reported by Balasar who found a large pericentric inversion of chromosome 1 (46, XY, inv (1) (p22q32)) during a routine chromosomal analysis [46].

LncRNAs are categorized into five subgroups depending on their different transcriptional forms: sense lncRNAs, antisense lncRNAs, intronic lncRNAs, intergenic lncRNAs, and divergent lncRNAs [47]. Intergenic and antisense lncRNAs constituted 23.2% and 48.1%, respectively, i.e., almost three-quarters of the identified DE lncRNAs. Antisense lncRNAs are transcribed from the antisense strand of the protein-coding genes, whereas intergenic lncRNAs are transcribed from both strands [47, 48]. Several abnormally expressed intergenic and antisense lncRNAs identified in patients with oligozoospermia indicated that lncRNAs regulated protein-coding genes during oligozoospermia. Moreover, intergenic lncRNAs have higher tissue specificity than protein-coding genes and thus are excellent indicators of different cell subsets in the auxiliary diagnosis of oligozoospermia [47, 49].

GO and KEGG analyses revealed several genes and pathways to be enriched in protein transport and localization, such as protein targeting to ER, SRP-dependent co-translational protein targeting to the membrane, and protein localization, in the OS group. Thus, we speculated these DE genes to be associated with spermatogenesis dysfunction and used them as markers for predicting oligozoospermia and studying its molecular mechanism. However, we ignored the fact that the degree of spermatozoa apoptosis was considerably higher in patients with oligozoospermia than in normal individuals [50], whereas hyperactive regulation of protein translation, transcription, and transport could stimulate cell apoptosis. Therefore, there existed differences in protein transport and localization between patients and normal individuals.

Endoplasmic reticulum stress occurs due to the accumulation of unfolded proteins caused by impaired protein folding or mutations [51]. Abnormal activation of protein targeting processes transport excess peptides to ER, thus increasing the probability of protein unfolding [52]. Three branches of ER stress response have been identified, namely IRE1 (inositol-requiring-1), PERK (protein kinase RNA-like ER kinase), and ATF6 (activating transcription factor-6) [53–56].

ER stress acts as a bridge between protein targeting and spermatozoa apoptosis. The enriched GO terms and KEGG pathways corresponded to negative regulation of ER stress response. Other ER stress-related GO terms, such as ER unfolded protein response (UPR), ATF6-mediated UPR, IRE1-mediated UPR, and PERK-mediated UPR were not differentially expressed. However, EIF2A, ATF4, EIF2AK1, and CEBPZ—vital genes in the PERK-EIF2α ER stress pathway—were differentially expressed. Limitations of RNA-sequencing could result in the inaccurate measurement of underexpressed genes, thus eliminating or underestimating certain ER-related underexpressed genes in differential expression analysis, and consequently interfering with GO and KEGG pathway analyses [57, 58]. Moreover, the current methods of GO and KEGG pathway analyses focus on the number of significant genes but ignore the participation of genes in different or opposite pathways.

PERK is a type-I transmembrane kinase located in the ER membrane that phosphorylates EIF2α [59, 60]. Phosphorylated EIF2a initiates the translation of poorly translated mRNAs such as ATF4 [59, 61]. ATF4, a transcription factor, controls redox homeostasis in the ER, and its translation results not only in amino acid biosynthesis, autophagy, protein folding, but also apoptosis via activation of the proapoptotic factor CEBPZ (growth arrest and DNA damage/CEBP homology protein, the major pro-apoptotic transcription factor induced by ER stress) [59, 62]. We found that EIF2A, ATF4, EIF2AK1, and CEBPZ were upregulated, indicating activation of the PERK-EIF2α axis and DNA damage-induced cell death.

Genes involved in the PERK-EIF2α pathway are also well-known activators of NF-κB signaling [51, 63], which is involved in the host’s innate immunity responses and prevention of apoptosis by repressing CEBPZ expression [51, 64]. However, we observed that both CEBPZ and NF-κB were upregulated, suggesting a complex mechanism of NF-κB-modulated gene expression and protein synthesis of CEBPZ. The IRE1-TRAF2 pathway can also activate NF-κB signaling, UPR-induced gene XBP1, and apoptosis-promoting factor p53 suppressor gene JNK1 [65, 66]. We observed that JNK1 was downregulated and XBP1 was upregulated; however, ERN1, the gene encoding TRAF2 and IRE1, was not expressed.

We also identified several pathways related to oxidative stress and neuronal apoptosis. Oxidative stress has been implicated in several neurodegenerative diseases, such as Alzheimer’s disease and Parkinson’s disease. Moreover, ER stress is known to trigger oxidative stress [67]. Thus, ER stress-triggered oxidative stress and consequent apoptosis in sperm cells could be responsible for the manifestations of oligozoospermia. Studies have reported a direct link between protein unfolding and the production of reactive oxygen species (ROS) [67–70]. Protein disulfide isomerase (PDI), endoplasmic reticulum oxidoreductin (ERO1), nuclear factor erythroid 2-related factors (Nrfs), and NADPH oxidases (NOXs) are major enzymatic components of ROS production during UPR [67–70]. Furthermore, CEBPZ induces ERO1 and PDI gene family to activate NOXs, consequently enhancing cytosolic ROS production [71, 72] and depleting antioxidants such as glutathione (GSH). We found that both ERO1-α and ERO1-β, and PDI genes, such as ERP29, PDIA2, PDIA5, TMX2, and TXNDC12, were upregulated in patients with oligozoospermia. Although we did not observe the expression of NOX family genes, the downstream genes of oxidative stress, including MAPK14, SP1, GPX1, and NOX5, were activated. These findings indicated that the PERK-EIF2α pathway triggered ER stress and interfered with ROS generation via the PDI-ERO1 cycle. In addition, PERK phosphorylated and activated transcription factor Nrf2, translocating it to the nucleus to activate the antioxidant response elements and maintain GSH levels [73]. The Nrf2 gene was not differentially expressed in patients with oligozoospermia, suggesting the sperm antioxidant stress response system could be blocked by unfolded proteins-induced pro-survival and pro-apoptotic signaling events.

The ER stress-induced pre-emptive quality control (ERpQC) can selectively degrade ER-targeting proteins by inhibiting their translocation via rerouting or degradation, thus limiting further protein loading into the stressed ER [51, 74–76]. During ER stress, the DERLIN family proteins, the ribosome nascent chain, and signal recognition particle complex (RNC-SRP) are rerouted from the ER translocation pathway to the cytosolic degradation pathway [52, 74–76]. Furthermore, the E3 ligase ubiquitinates the fully translated proteins and effectively transports them to the proteasome by p97 and Bag6 chaperones for degradation by the ER-associated degradation system (ERAD) [77–79]. Several proteins involved in the organization of the retro-translocon channel, such as Sec61, Derlin family proteins, p97, and HRD1 E3 ligase, have been described [74–79]. However, genes encoding these proteins were not expressed in patients with oligozoospermia under PERK-EIF2α pathway-induced ER stress. This finding suggests that the ERpQC system could become dysfunctional to protect the sperms under ER stress, resulting in conformational diseases due to accumulation of unfolded proteins.

GO and KEGG pathway analyses indicated that DE genes, such as SRP9, EIF2A, CEBPZ, EIF2AK3, and NFKB1, were mainly enriched in protein targeting, ER stress, and oxidative stress. LncRNAs are transcribed along with the co-expressed genes they regulate [47]. Therefore, the functions of lncRNAs in oligozoospermia could be studied by the function of related target genes. Their cis or trans regulation by lncRNAs, such as EIF2A trans-regulator lnc-FAM198B-1, could regulate spermatogenesis and sperm apoptosis-associated pathways and can be considered as potential diagnostic or therapeutic targets.

Some of the previously reported genes related to spermatogenesis disorders, such as the SOX gene family, SPATA gene family, and TAM receptor genes, were differentially expressed in our study. Consistent with the findings of a study by Jiang who reported that the Sox gene family maintains adult male fertility [80], our study found that reduced expression of SOX genes blocked the initiation or progression of spermatogenesis and affected cell maturation. Furthermore, SPATA family genes function in spermatogenesis, sperm maturation, and fertilization. Hypermethylation in the promoter regions of SPATA family genes correlates with oligozoospermia and male infertility [81]. Although SPATS2 and SPATA21 were downregulated in patients with oligozoospermia in our study, altered methylation patterns were not reported. Lu reported that mice knockout for genes (TYRO3, AXL, and MER) were deficient in sperm maturation [82].

## CONCLUSIONS

**Figure 9:**
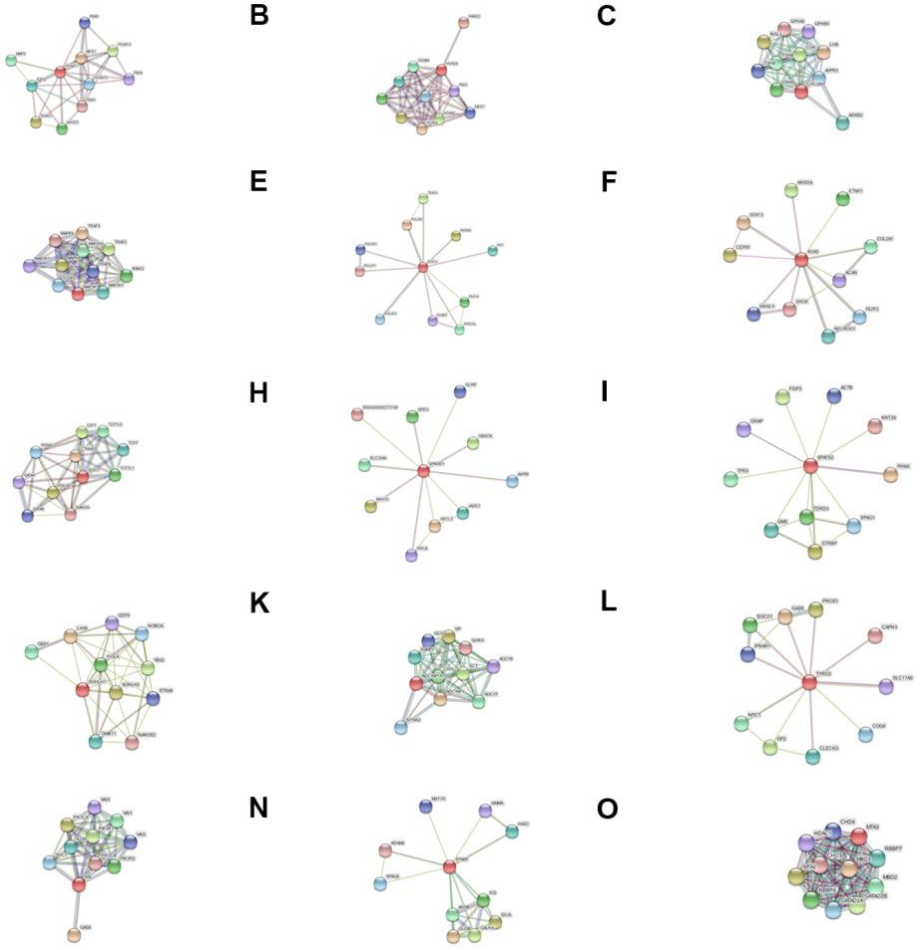
Protein-protein (PPI) interaction networks for (**A**) HOXA11, (**B**) HOXD9, (**C**) LHCGR, (**D**) MAP3K1, (**E**) SOX12, (**F**) SOX5, (**G**) SOX17, (**H**) SPATA21, (**I**) SPATS2, (**J**) SOHLH1, (**K**) ADCYAP1R1, (**L**) TYRO3, (**M**) AXL, (**N**) SPAM1, and (**O**) MTA3.

We identified key lncRNAs, pathways, and gene sub-networks using RNA sequencing datasets that are associated with the pathogenesis of oligozoospermia (Figure 9). The identified lncRNAs and pathways could serve as effective therapeutic targets for male infertility.

## MATERIALS AND METHODS

### Sperm collection and experimental design

All 12 human sperm samples were obtained from Peking University International Hospital from March 2016 to December 2016. This study was approved by the Ethics Committees of Peking University International Hospital and was conducted in accordance with the Declaration of Helsinki. Participants provided their informed consent in writing. Participants were selected by excluding those with abnormal karyotype and Y chromosome microdeletion. The clinical data of all participants are shown in Table 1.

### Collection, analysis, and purification of sperm samples

All human sperm samples were collected and analyzed according to the WHO guidelines using a Makler R chamber (Sefi Laboratories, Tel Aviv, Israel) [17]. Normozoospermic samples were defined as those with concentrations ≥ 15 × 10^6^/mL. Oligozoospermic samples had a concentration of < 15 × 10^6^/mL. All sperm samples were cryopreserved at 1:1 dilution (Irvine Scientific, Santa Ana, CA, USA) in 2 mL cryogenic vials to check the yolk buffer and equilibrated to room temperature for 15 min. Next, all sperm samples were suspended 10 cm above liquid nitrogen for 1 to 2 h and finally transferred to liquid nitrogen. All samples were thawed as follows: cryotubes were transferred to a 37° C water bath and incubated for 5 min. The cell suspension was centrifuged at 500 g for 5 min and the supernatant was discarded. Sperms were purified and the RNA was extracted immediately. The samples were stored at −80° C until further processing.

### RNA extraction and quality control

The RNA was extracted from frozen human sperm samples using an RNA Extraction Kit (Qiagen, Hilden, Germany) according to the manufacturer’s protocol. The RNA was quantified by NanoDrop ND-1000, and the RNA quality was assessed on Agilent 2100 Bioanalyzer (Aligent Technologies, Santa Clara, CA, USA). The RNA integrity was assessed by running the sample on a standard denaturing agarose gel.

### Library preparation and lncRNA sequencing

Total RNA was isolated using the Trizol reagent (Invitrogen, CA, USA). The RNA quantity and purity were detected by Bioanalyzer 2100 and RNA 6000 Nano LabChip Kit (Agilent Technologies). High-quality RNA (10 μg; RNA integrity number > 7) per sample was obtained by removing ribosomal RNA using the Ribo-Zero Gold rRNA removal kit (Illumina, San Diego, USA) according to manufacturer’s instructions. Approximately 1.5 μg of purified RNA per sample was used to construct the sequencing libraries using the NEBNext Ultra Directional RNA Library Prep Kit from Illumina (NEB, Ipswich, MA, USA) and index codes were added to the corresponding samples. Briefly, the purified RNA was fragmented into small pieces using divalent cations at elevated temperatures. The first-strand cDNA was synthesized using cleaved RNA fragments as templates, random hexamer primers, and M-MuLV reverse transcriptase. Subsequently, the second-strand cDNA was synthesized using Polymerase I and RNase H with reaction buffer (dTTPs were replaced by dUTPs). Exonuclease/polymerase was used to degrade the remaining overhangs and create blunt ends. After adenylation of the 3’ ends of DNA fragments, NEBNext Adaptor with a hairpin loop structure was ligated for hybridization. The 300-bp DNA fragments were selected and purified with AMPure XP system (Beckman Coulter, Beverly, MA, USA). Paired-end sequencing was performed on an Illumina HiSeq X-Ten (Novogene Corporation, Beijing, China).

### Identification of lncRNAs and mRNAs

To obtain high-quality lncRNA and mRNA reads, the adaptor sequences and low-quality sequences (N bases > 10% or base quality < 10; greater than 50%) were removed. The remaining reads were aligned to the UCSC (human genome, hg19) using HISAT (v0.1.6). The mapped reads were assembled by StringTie (v.1.0.4) separately. Next, all assembled transcripts from samples were merged to reconstruct a comprehensive transcriptome with Cuffcompare (v.2.1.1). To obtain highly reliable novel lncRNAs, we first removed the transcripts with a length of less than 200 bp. Transcripts with peak expression of less than 2.0 across all samples and present only in one sample were considered background and removed [18]. The transcripts overlapping with known RNAs (lncRNAs and mRNAs) on the same strand were removed. The remaining transcripts encoding any conserved protein domains were excluded by Pfam database alignment with HMMER. Finally, the transcripts predicted by the lcRNA prediction software (CPC v0.9-r2 and CNCI v2.1) were used for further analysis.

### Analysis of DE lncRNAs and DE mRNAs

HTseq (v0.6.1) feature extraction software was used to calculate the number of readings of each gene (and lncRNA). The R software Limma package was used to normalize the expression data. Genes with low expression were eliminated by filterByExpr function in edgeR package with parameters: min.count = 0, min.total.count = 15, large.n = 10, min.prop = 0.7. Raw counts were converted to log2 count per million (CPM) reads. Next, we checked the outliers of the standardized data, calculated the count outside the threshold using the mean and standard deviation method (× ± 2 SD) as the outliers, and replaced the count with the predicted value of the trimmed average of all samples. This method is similar to replacing outliers in the DESeq2 package using Cook’s distances. Subsequently, the limFit function of the Gaussian regression model in the Limma package was used for model fitting, and DE genes (and lncRNAs) in RNA sequencing data were identified using the empirical Bayes estimation. The regression model was adjusted for age and BMI that could interfere with the analysis. The corrected *p*-value (corrp) was calculated based on the Benjamini–Hochberg controlled error detection rate (FDR) [1]. The thresholds of DE genes and lncRNAs were corrected as *p*-value < 0.05 and fold change ≥ 2, respectively.

### GO and KEGG enrichment analyses

To study the biological effects of DE lncRNAs in sperm samples, over-representation analysis method (ora) of R software gene ontology for RNA-seq package (goseq, v1.38.0) and the pathway level analysis method (plage) of gene set variation analysis package (gsva, v1.34.0) were used to determine GO terms and KEGG pathways. Based on GO and KEGG databases, these functions mapped the target genes to the corresponding pathways or biochemical processes. The *p*-value was calculated to indicate the significance of gene enrichment. For both GO and pathway analyses, a *p*-value < 0.05 was considered significant.

### Co-expression network analysis

Compared with the traditional method of constructing a co-expression network using Pearson’s correlation coefficient, the Gaussian graphical model (GGM) and weighted correlation network analysis (WGCNA) efficiently removed the pseudo correlation effect on the network structure. We used the R software high-throughput Gauss map model package (Statistical Inference of Large-Scale Gaussian Graphical Model [SILGGM]) to construct the co-expression network of mRNAs and lncRNAs. The identified co-expressed mRNAs and lncRNAs (*p*-value < 0.05) consisted of strong co-expression pairs with a partial correlation coefficient 1 ≥ *r* > 0.95 or –1 ≤ *r* < –0.95, moderate co-expression pairs with a partial correlation coefficient 0.95 ≥ *r* > 0.80 or –0.95 ≤ *r* < –0.80, and weak co-expression pairs with a partial correlation coefficient 0.8 ≥ *r* or –0.8 ≤ *r*. Further, we classified the co-expressed lncRNAs with a partial correlation coefficient > 0.95 or < – 0.95 through cis-trans analysis. LncRNAs having flanked genes within 300 kb were considered as cis-acting lncRNAs, and those with lncRNAs-gene pair sequence homology (blastn, E-value < 1.0E-10 and identity > 99 and matched length ≥ 20 bp) were considered as trans-acting lncRNAs.

### QPCR analysis

Isolated RNA was reverse-transcribed to cDNA using a Reverse Transcription Kit (Takara, Dalian, China). The qRT-PCR analyses were performed using a StepOnePlus RT-PCR Instrument with Power SYBR Green (Takara, Dalian China). The qRT-PCR conditions were as follows: 95° C for 2 minutes, followed by 40 cycles of 95° C for 15 seconds and 60° C for 30 seconds. All experiments were performed and analyzed in triplicate. The primers used in this study were listed in Table 1. Then, lncRNA expression levels were normalized to GAPDH and calculated using the 2−ΔΔCt method.

### Statistical analysis

Data are expressed as mean ± standard error of the mean (SEM). The *p*-values < 0.05 were considered significant. Graphs were plotted and analyzed using GraphPad Prism version 6.0 (GraphPad Software, La Jolla, CA, USA).

### Availability of data and materials

Data generated during the study and presented in the manuscript are available from the corresponding author upon request.

### Ethics approval and consent to participate

All experiments involving human sperm samples in this study were approved by the Ethics Committees of Peking University International Hospital. The participants provided their informed consent in writing.

## Supplementary Material

Supplementary Table 1

Supplementary Table 2

Supplementary Table 3

Supplementary Table 4

Supplementary Table 5

Supplementary Table 6

Supplementary Table 7

Supplementary Table 8

Supplementary Table 9

Supplementary Table 10

Supplementary Table 11

Supplementary Table 12

Supplementary Table 13

Supplementary Table 14

Supplementary Table 15

Supplementary Table 16

Supplementary Table 17

Supplementary Table 18

Supplementary Table 19

Supplementary Table 20

Supplementary Table 21

Supplementary Table 22
