# Supplementary material for: LncRNAs induce oxidative stress and spermatogenesis by regulating endoplasmic reticulum genes and pathways
Source: Aging (Albany NY). 2021 May 6;13(10):13764–87. doi: 10.18632/aging.202971 (PMC8202879; doi:10.18632/aging.202971)
Supplement: Supplementary Table 6 [file aging-13-202971-s006.pdf]

SUPPLEMENTARY TABLE

Supplementary Table 6. Group_lnc_condition.

Group_lnc	Percentage
Antisense Down	0.025381
Antisense Up	0.455584
Intergenic Down	0.098562
Intergenic Up	0.133249
Intron Down	0.021574
Intron Up	0.084179
Sense Down	0.050761
Sense Up	0.130711
