# Supplementary material for: LncRNAs induce oxidative stress and spermatogenesis by regulating endoplasmic reticulum genes and pathways
Source: Aging (Albany NY). 2021 May 6;13(10):13764–87. doi: 10.18632/aging.202971 (PMC8202879; doi:10.18632/aging.202971)
Supplement: Supplementary Table 20 [file aging-13-202971-s020.docx]

**Supplementary Table 20. TF factor.**

lnc-GNS-3


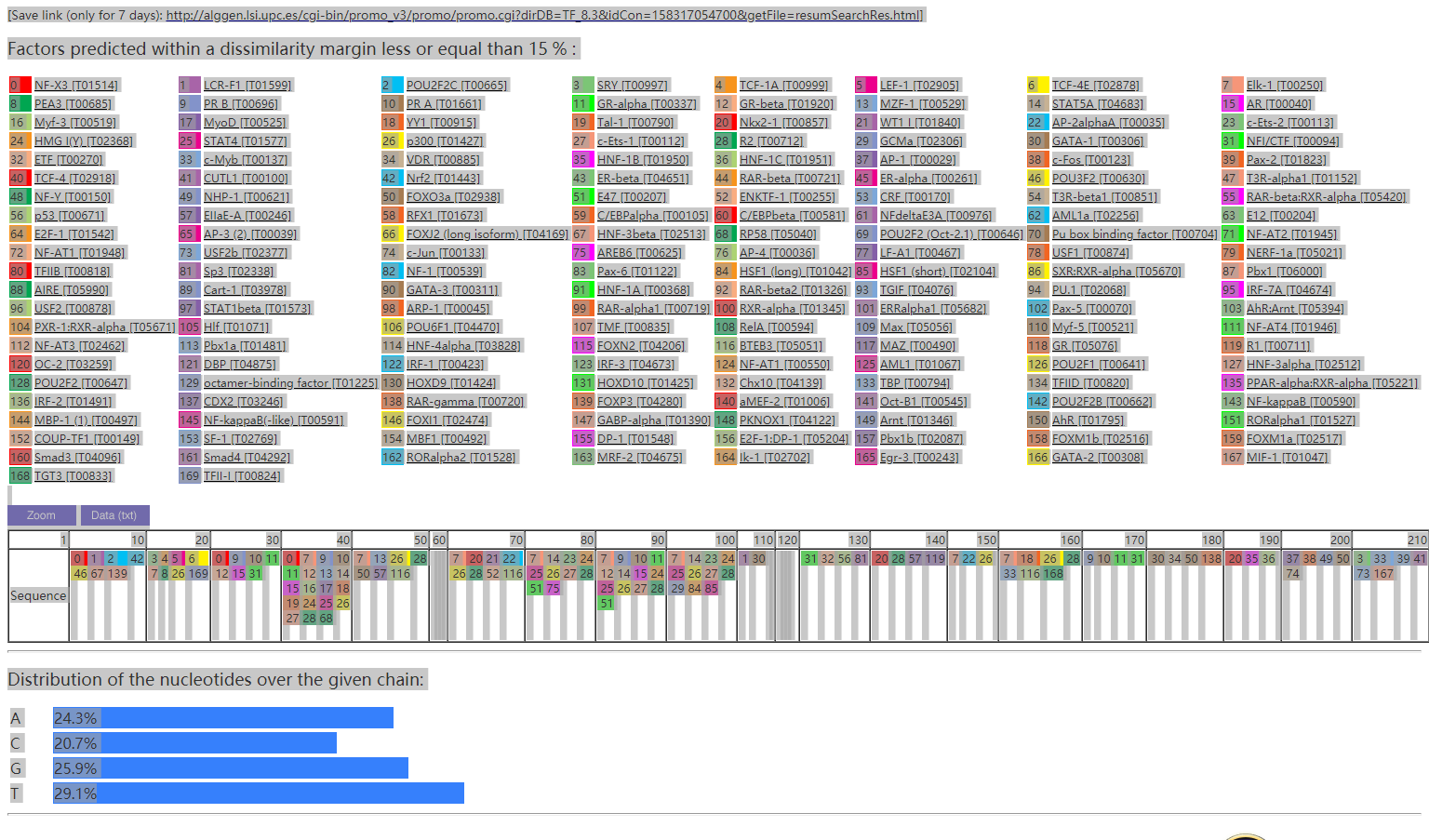


<http://alggen.lsi.upc.es/cgi-bin/promo_v3/promo/promo.cgi?dirDB=TF_8.3&idCon=158317054700&getFile=resumSearchRes.html>

XLOC_515910

lnc-SERHL2-7

lnc-IL31RA-1


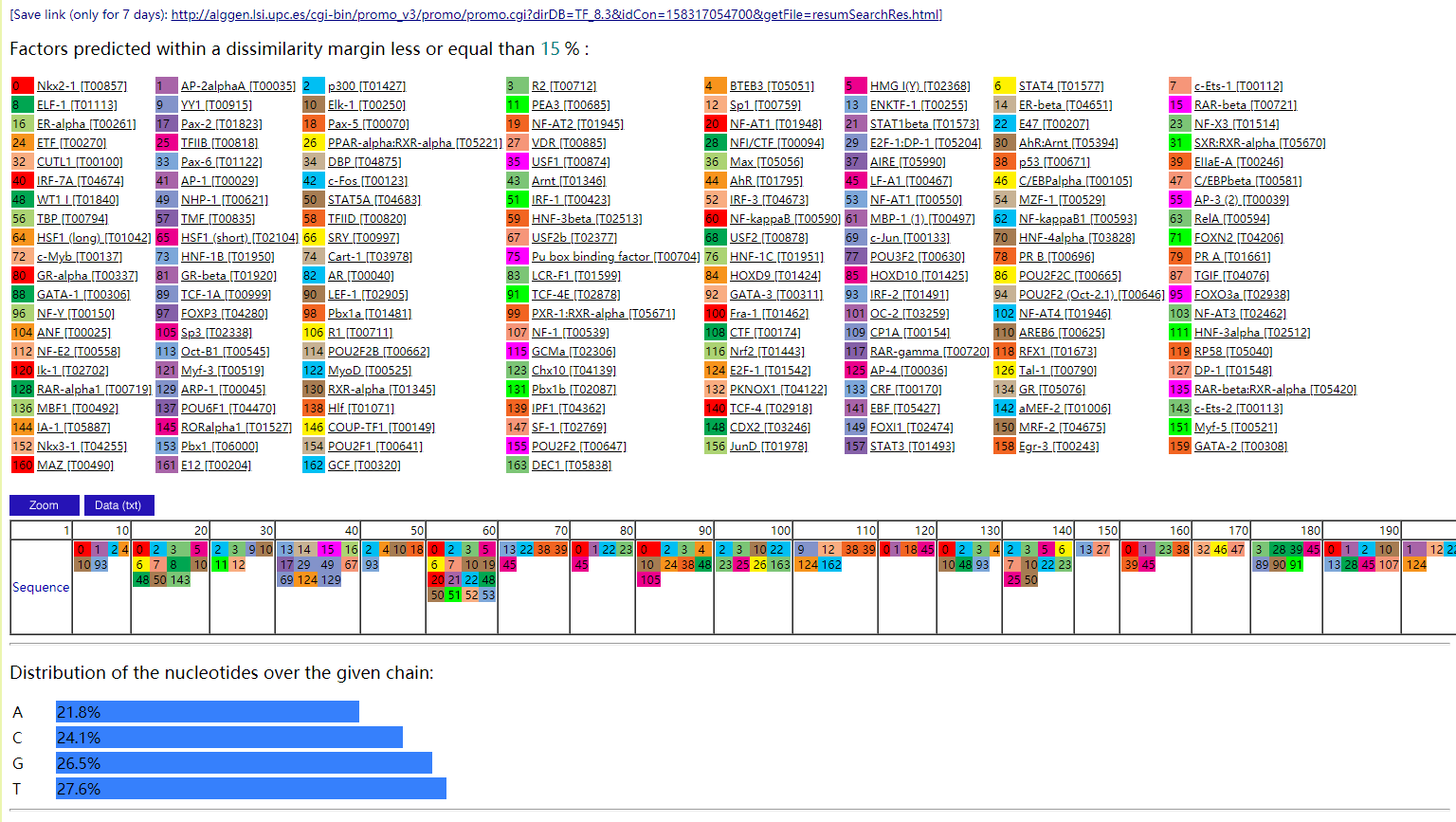


<http://alggen.lsi.upc.es/cgi-bin/promo_v3/promo/promo.cgi?dirDB=TF_8.3&idCon=158317054700&getFile=resumSearchRes.html>

lnc-NLRP2-2

lnc-CDK12-2


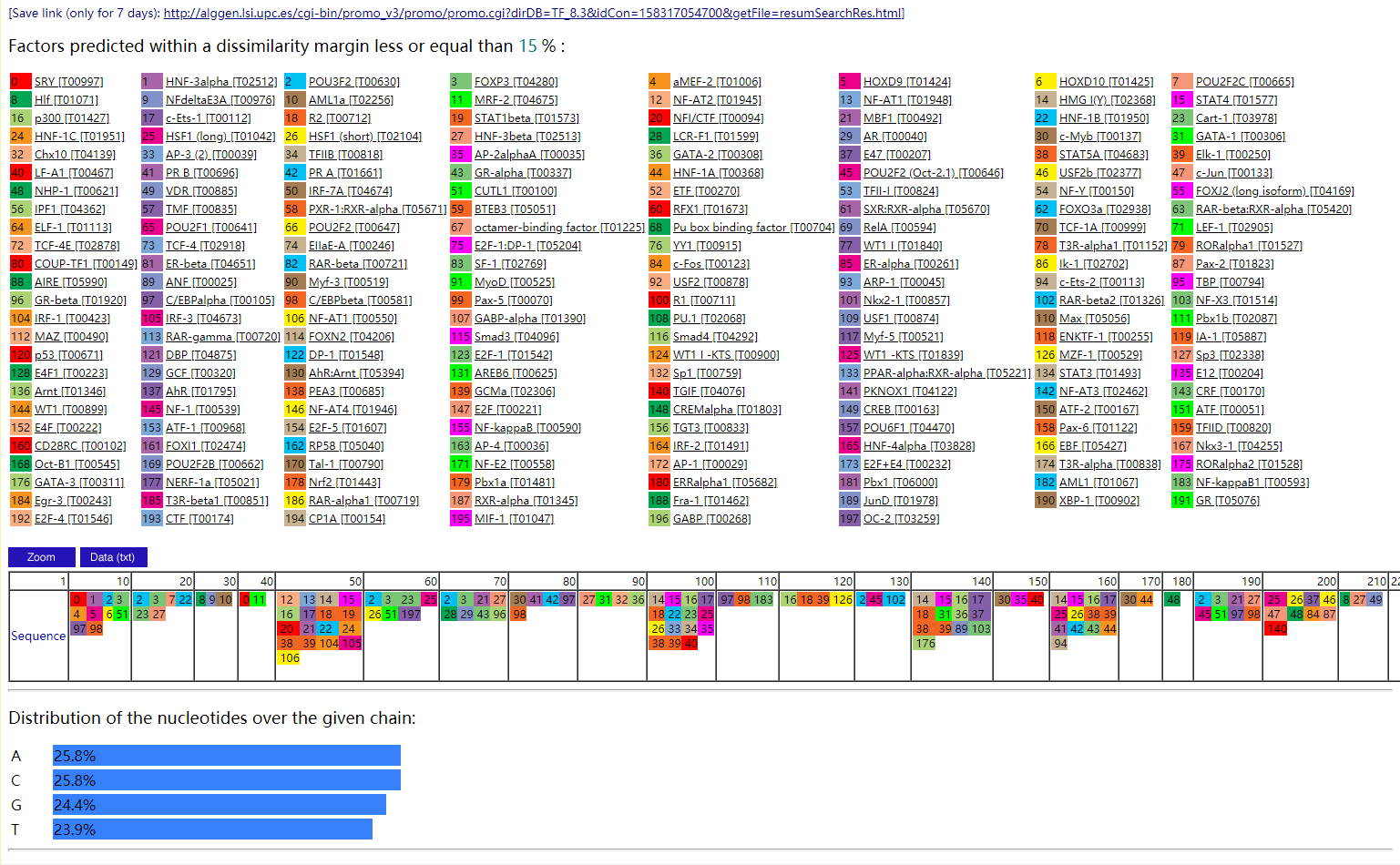


<http://alggen.lsi.upc.es/cgi-bin/promo_v3/promo/promo.cgi?dirDB=TF_8.3&idCon=158317054700&getFile=resumSearchRes.html>

XLOC_1093926

lnc-LRRC38-2


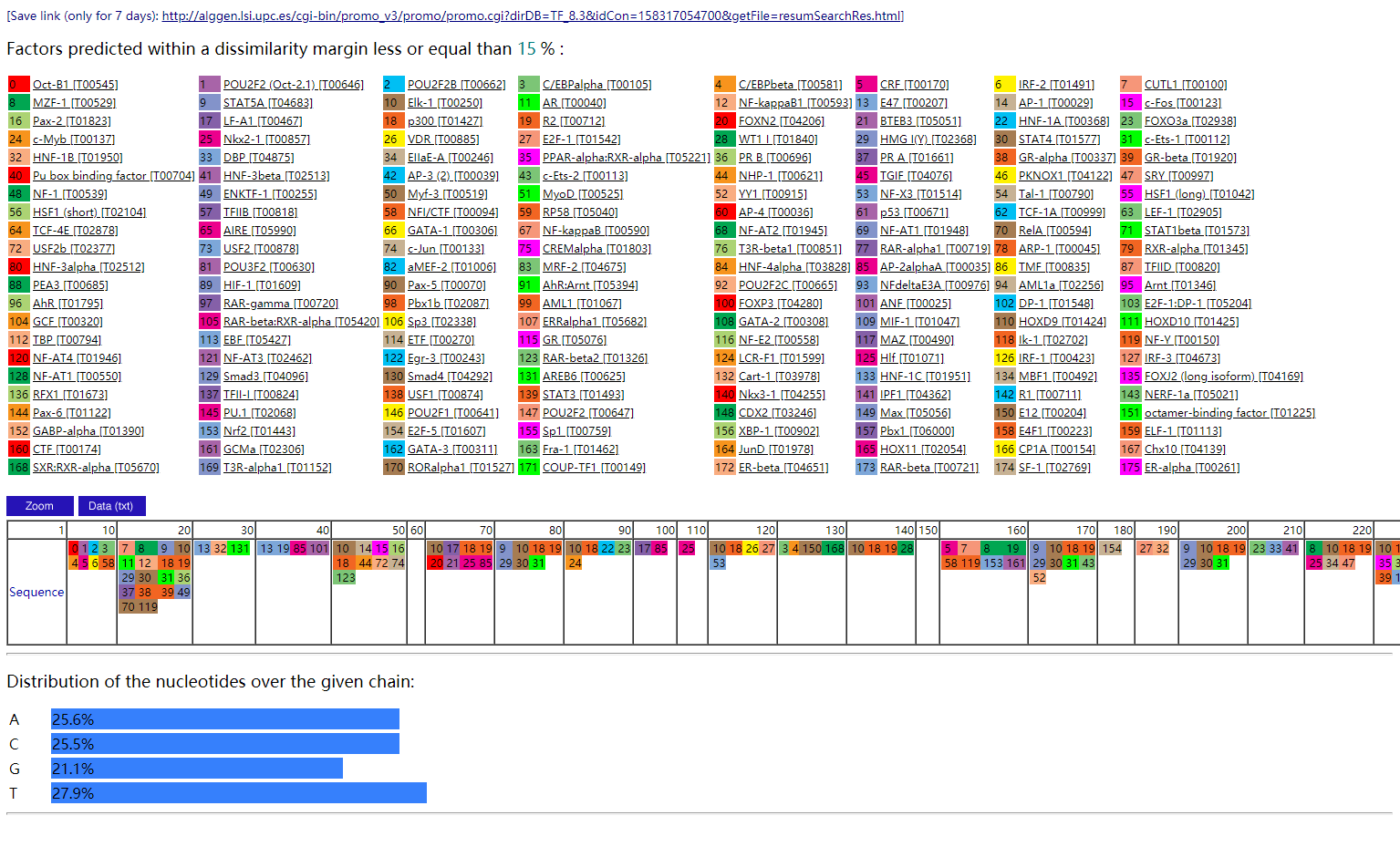


<http://alggen.lsi.upc.es/cgi-bin/promo_v3/promo/promo.cgi?dirDB=TF_8.3&idCon=158317054700&getFile=resumSearchRes.html>

lnc-FOXN1-1

lnc-SLC46A2-1


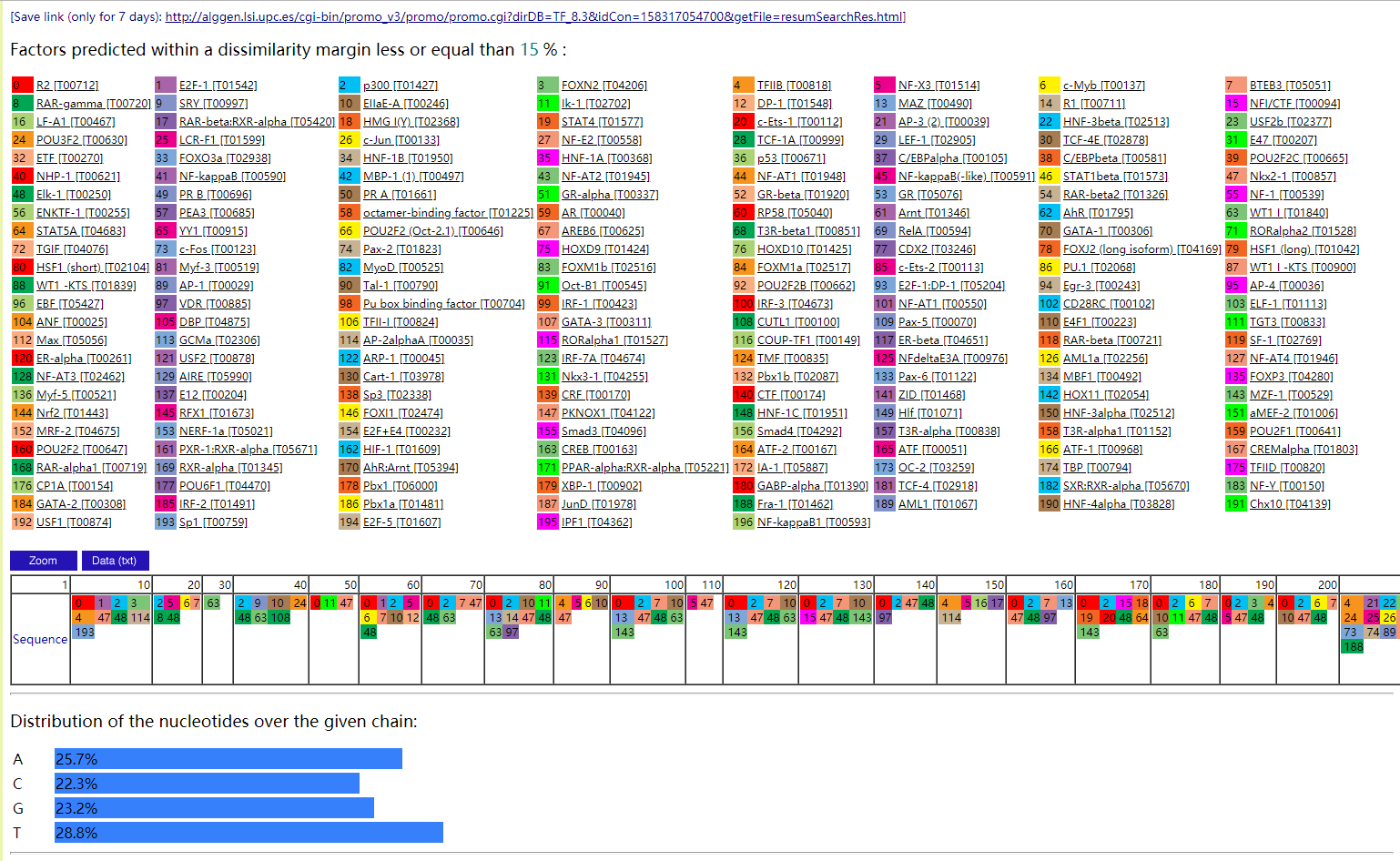


<http://alggen.lsi.upc.es/cgi-bin/promo_v3/promo/promo.cgi?dirDB=TF_8.3&idCon=158317054700&getFile=resumSearchRes.html>

lnc-CSNK1A1-7


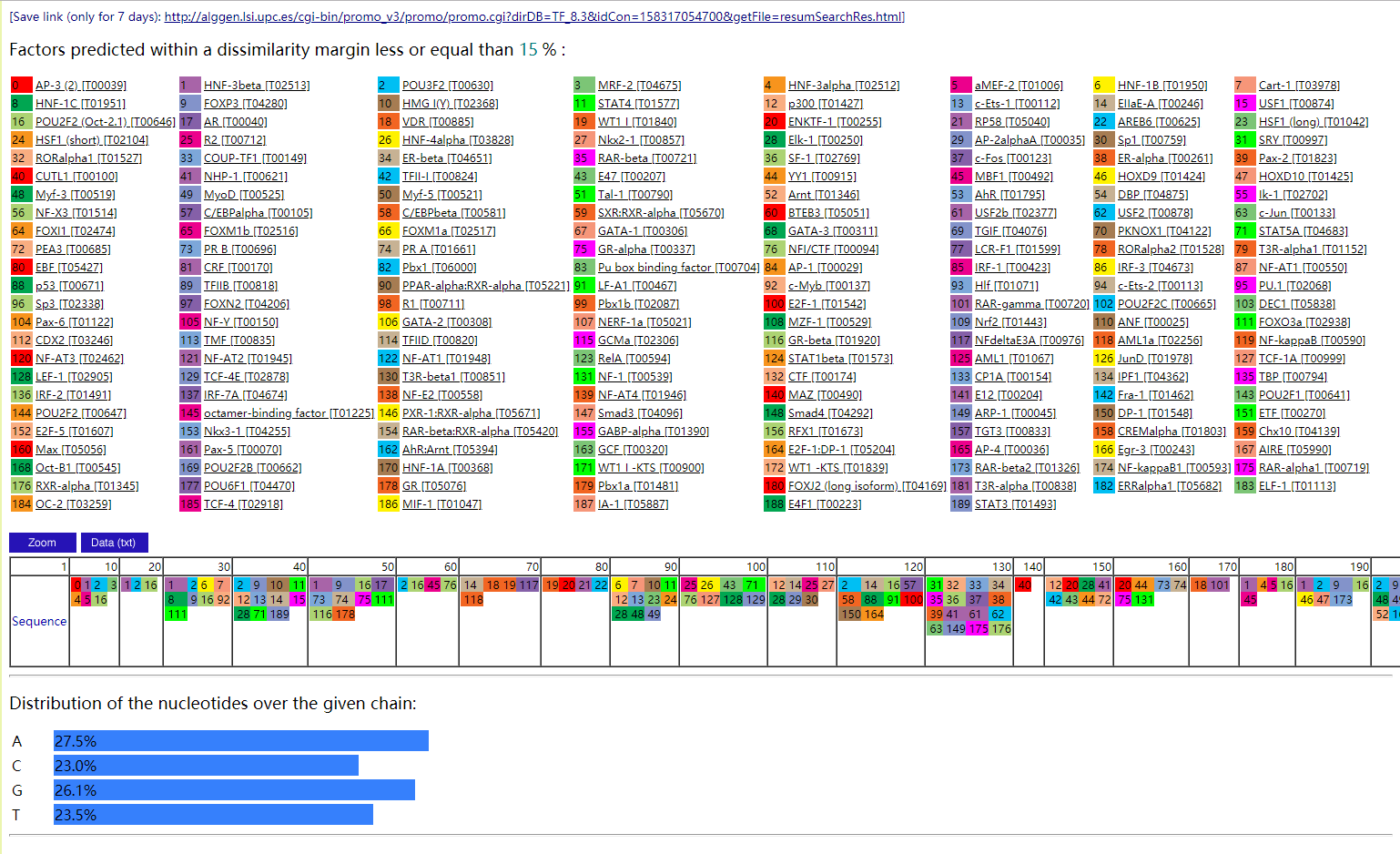


<http://alggen.lsi.upc.es/cgi-bin/promo_v3/promo/promo.cgi?dirDB=TF_8.3&idCon=158317054700&getFile=resumSearchRes.html>

lnc-PHLDB1-1

lnc-KB-1980E6.3.1-6

lnc-LAT2-2


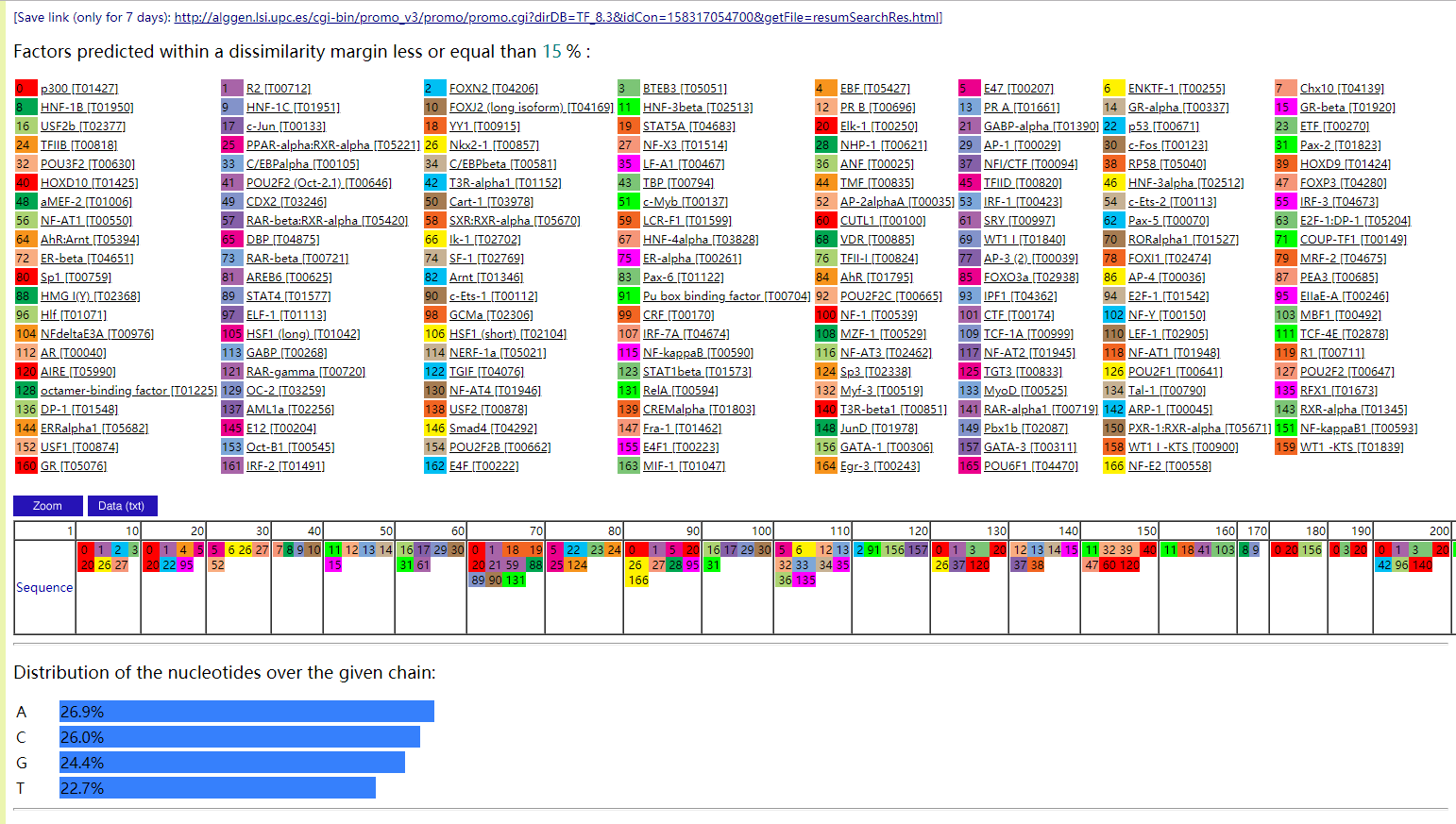


<http://alggen.lsi.upc.es/cgi-bin/promo_v3/promo/promo.cgi?dirDB=TF_8.3&idCon=158317054700&getFile=resumSearchRes.html>

XLOC_2394941

lnc-EEF1B2-3


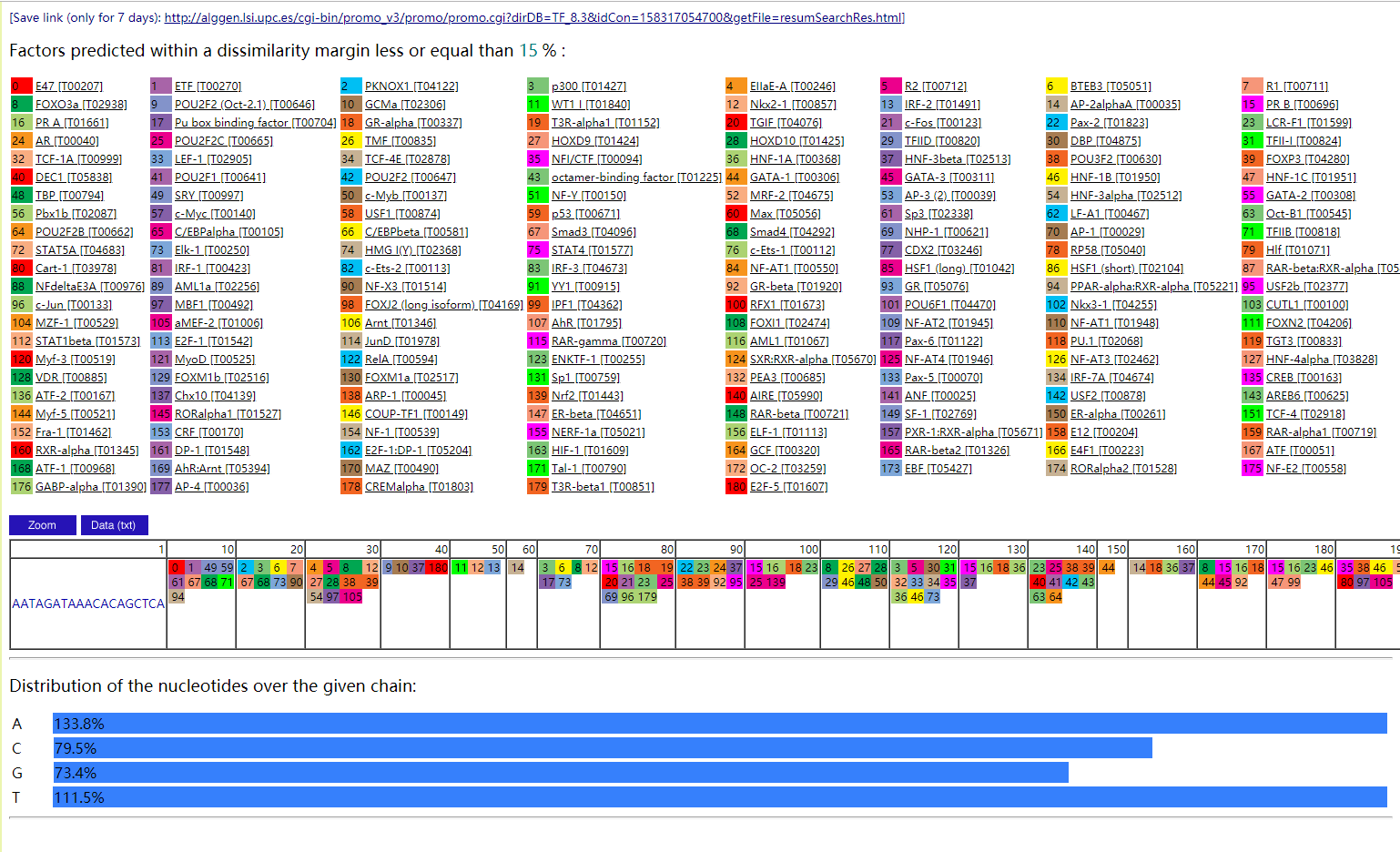


<http://alggen.lsi.upc.es/cgi-bin/promo_v3/promo/promo.cgi?dirDB=TF_8.3&idCon=158317054700&getFile=resumSearchRes.html>

lnc-AP3S1-15


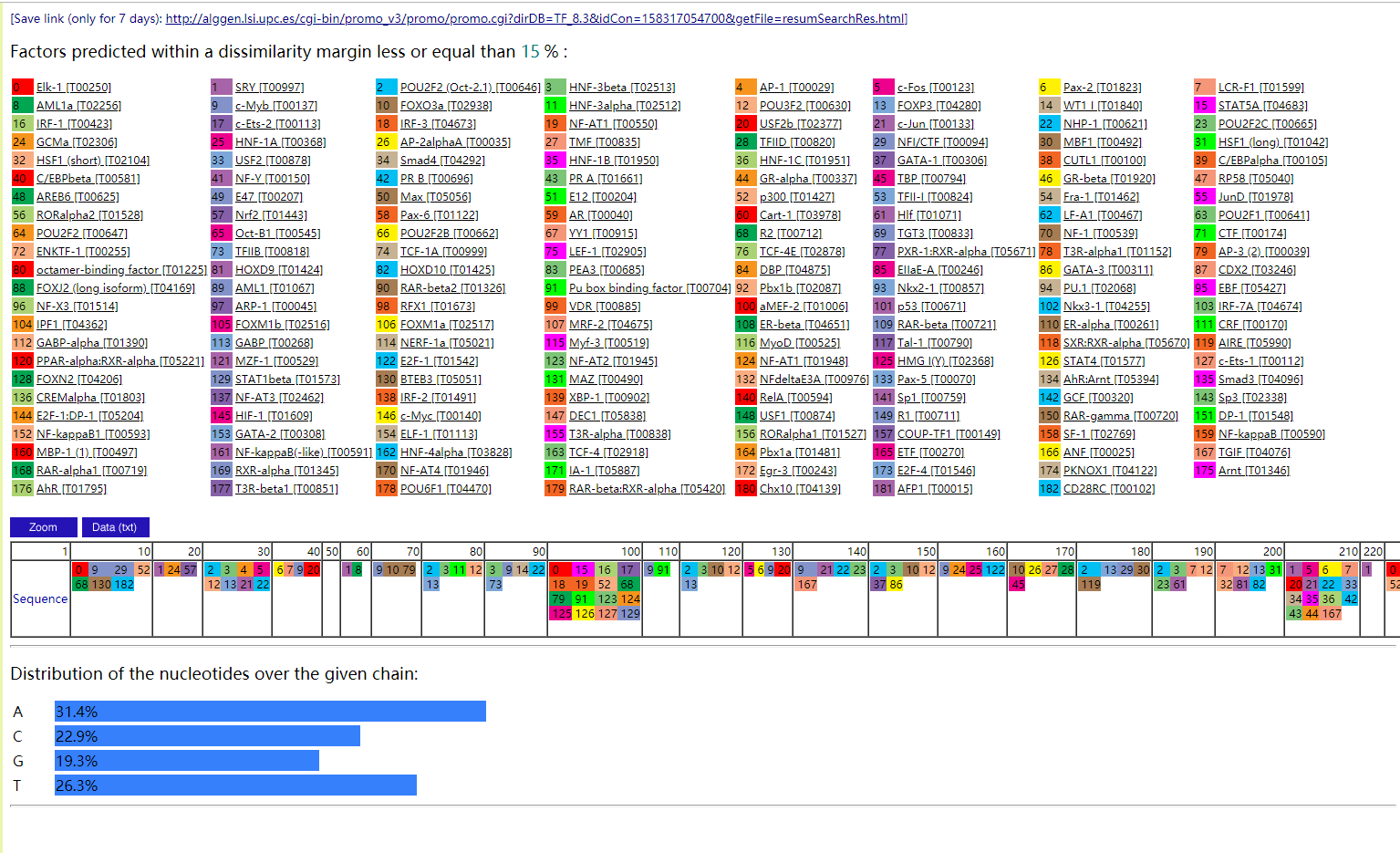


<http://alggen.lsi.upc.es/cgi-bin/promo_v3/promo/promo.cgi?dirDB=TF_8.3&idCon=158317054700&getFile=resumSearchRes.html>

lnc-SLA2-1

lnc-RAPH1-6


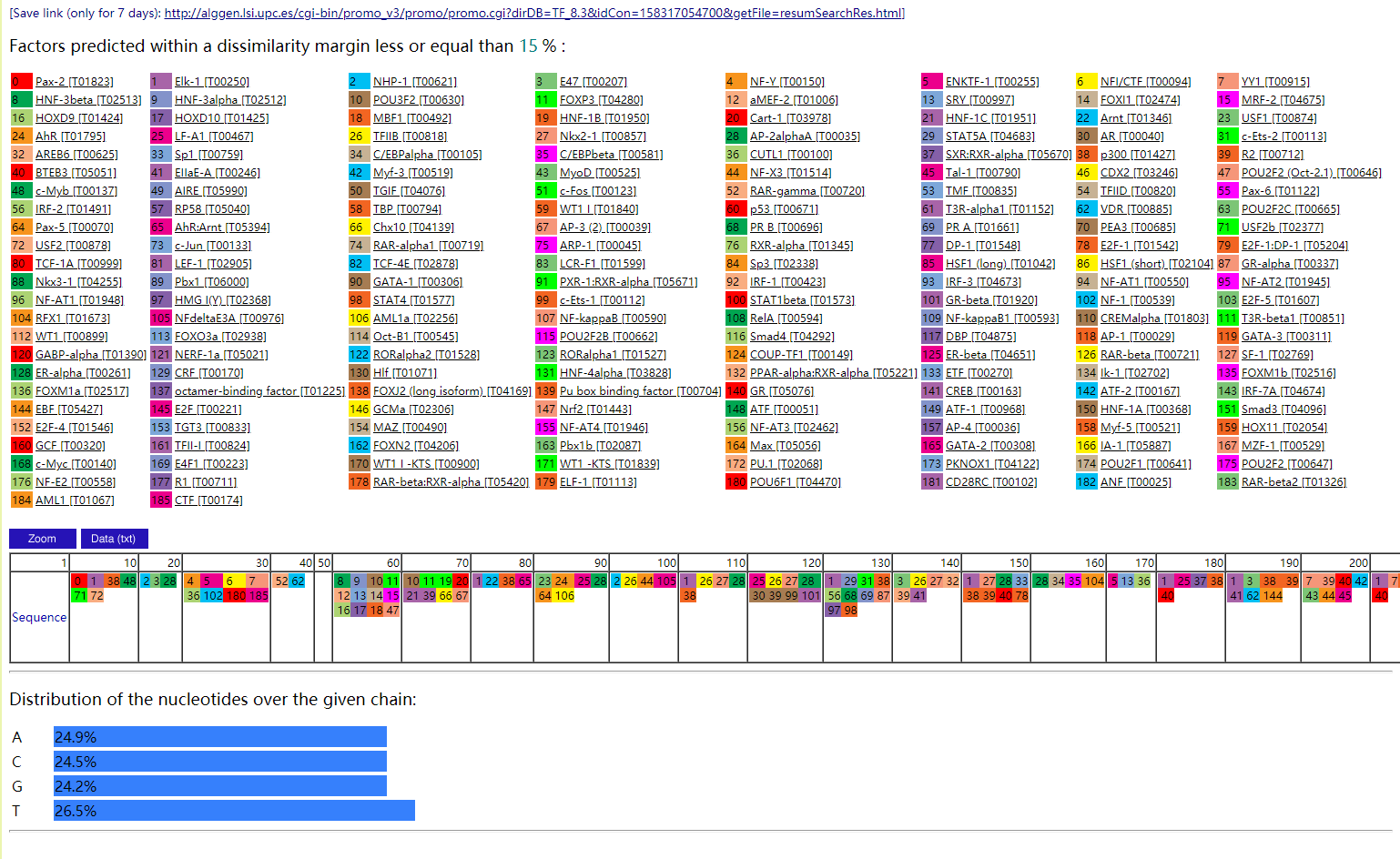


<http://alggen.lsi.upc.es/cgi-bin/promo_v3/promo/promo.cgi?dirDB=TF_8.3&idCon=158317054700&getFile=resumSearchRes.html>

lnc-TICAM1-1


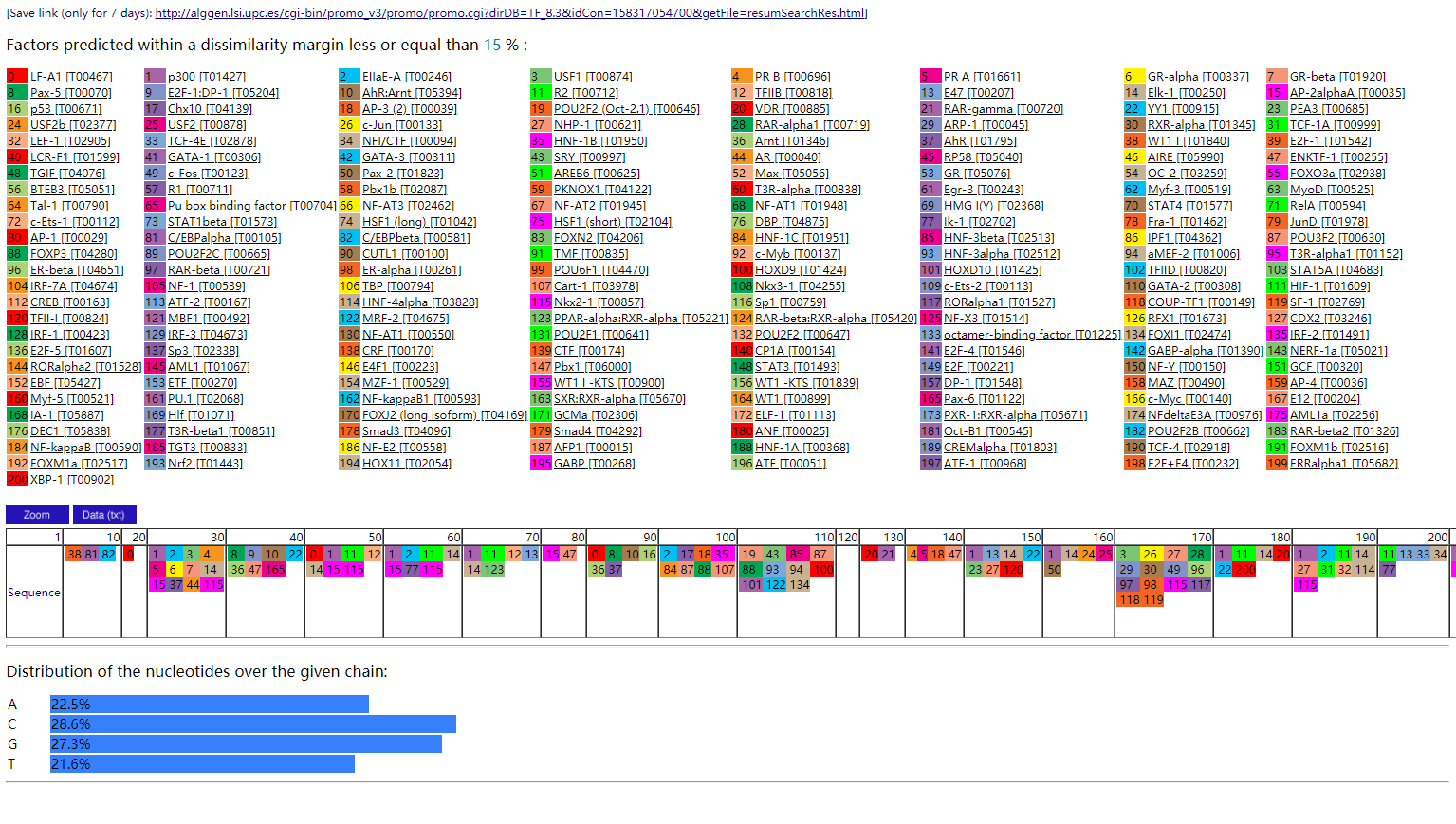


<http://alggen.lsi.upc.es/cgi-bin/promo_v3/promo/promo.cgi?dirDB=TF_8.3&idCon=158317054700&getFile=resumSearchRes.html>

lnc-AC007390.5.1-4

lnc-TSKS-1


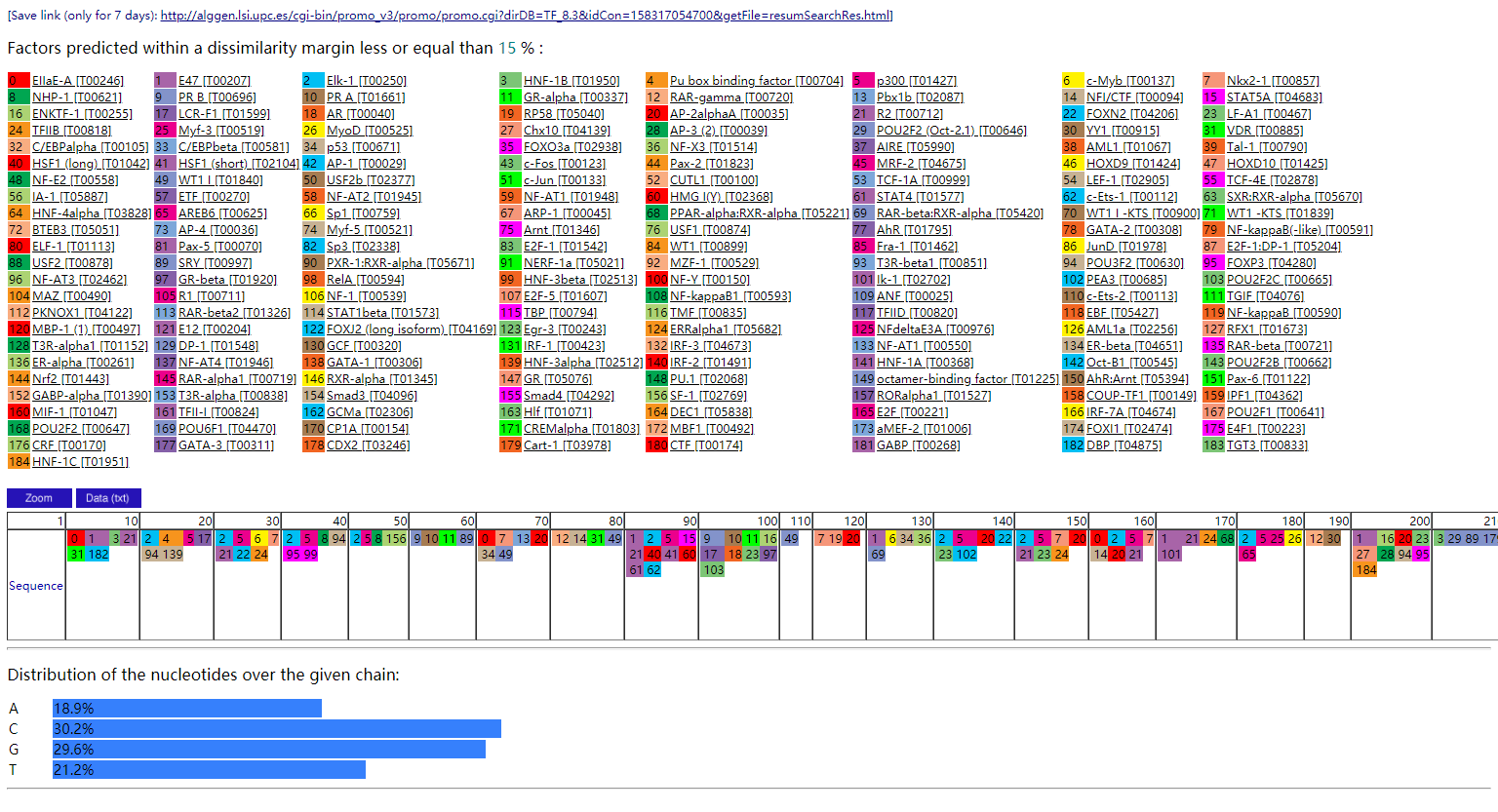


<http://alggen.lsi.upc.es/cgi-bin/promo_v3/promo/promo.cgi?dirDB=TF_8.3&idCon=158317054700&getFile=resumSearchRes.html>

lnc-TUFM-1


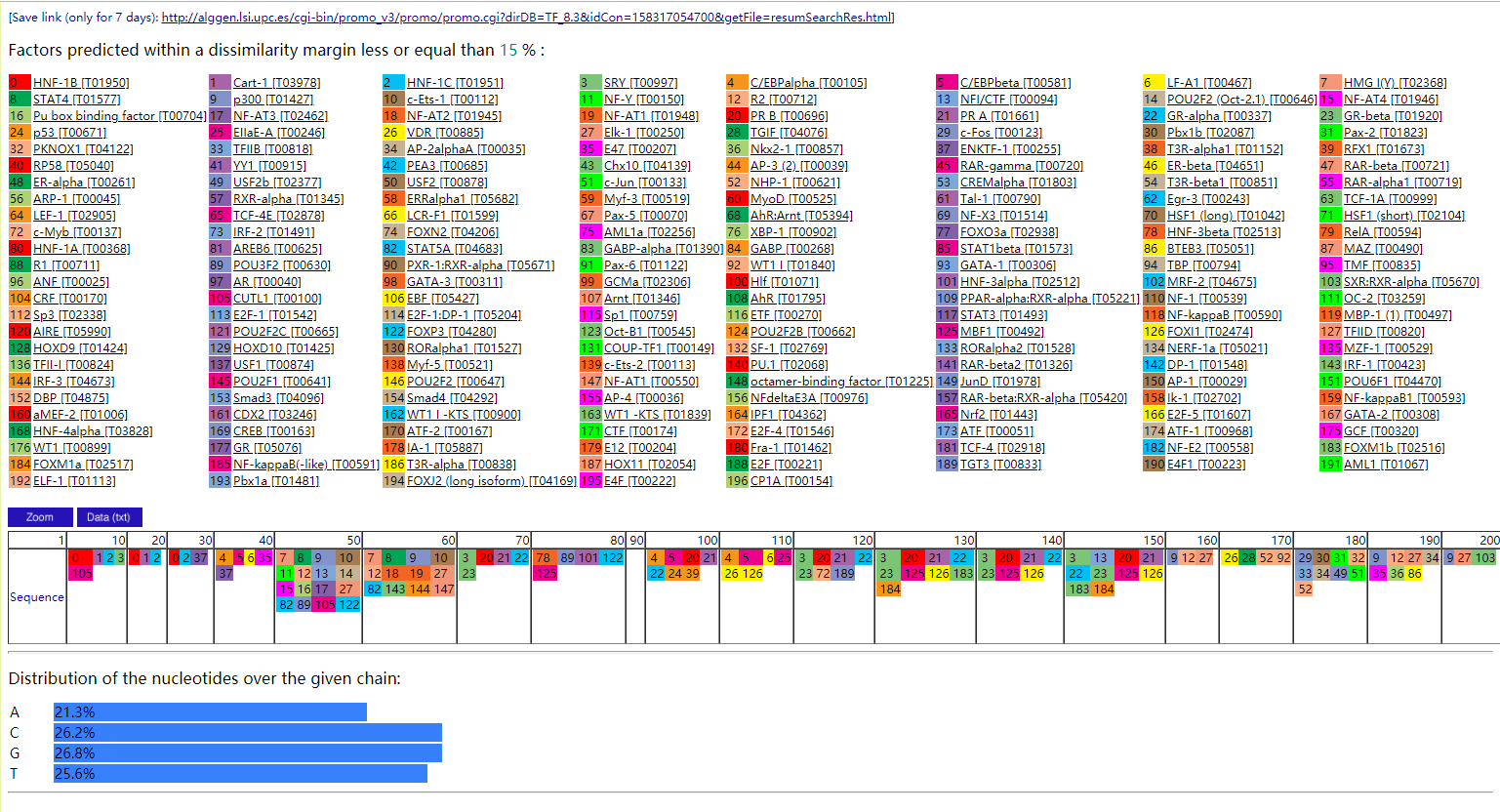


<http://alggen.lsi.upc.es/cgi-bin/promo_v3/promo/promo.cgi?dirDB=TF_8.3&idCon=158317054700&getFile=resumSearchRes.html>

lnc-VRK3-2


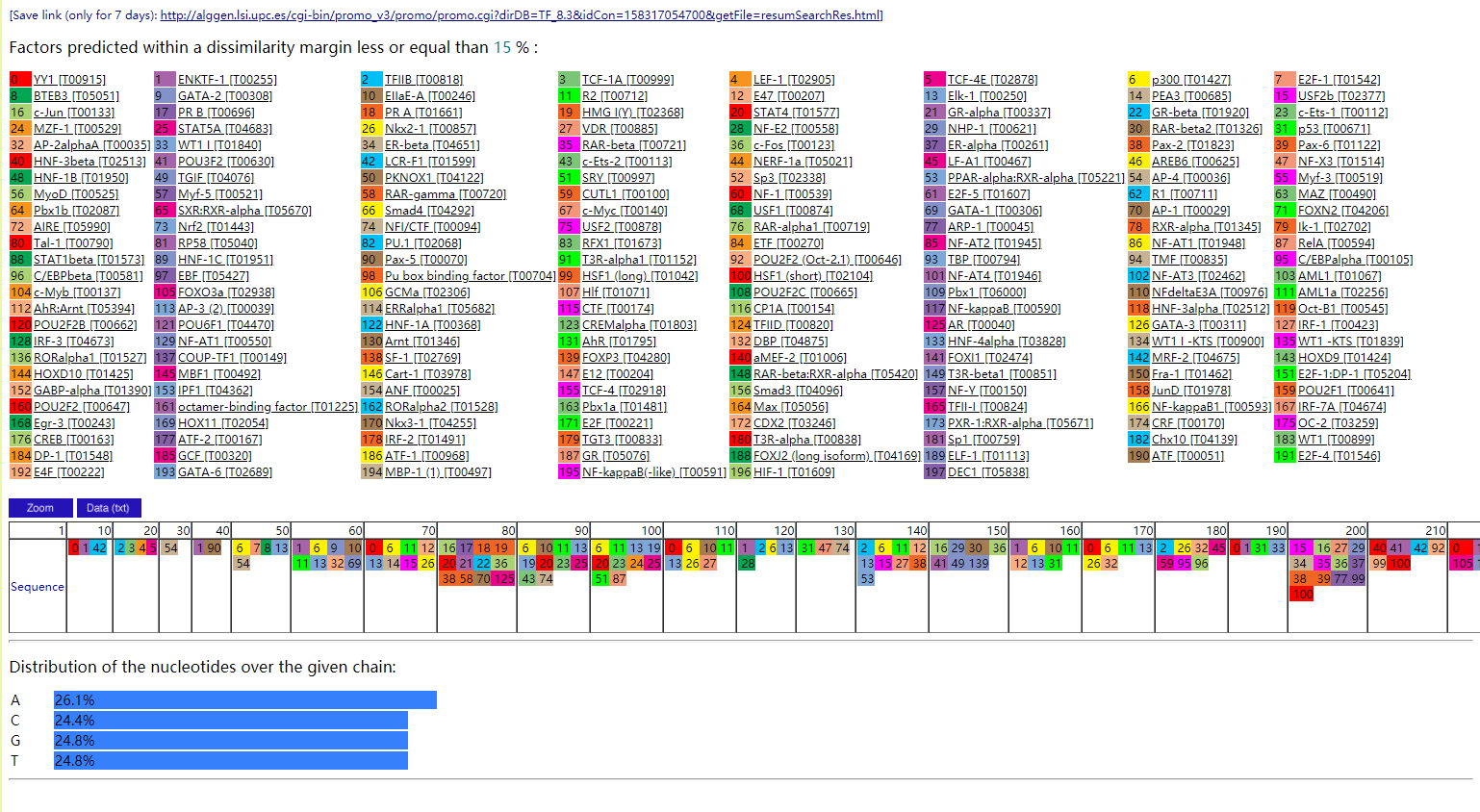


<http://alggen.lsi.upc.es/cgi-bin/promo_v3/promo/promo.cgi?dirDB=TF_8.3&idCon=158317054700&getFile=resumSearchRes.html>

lnc-SPATA21-3


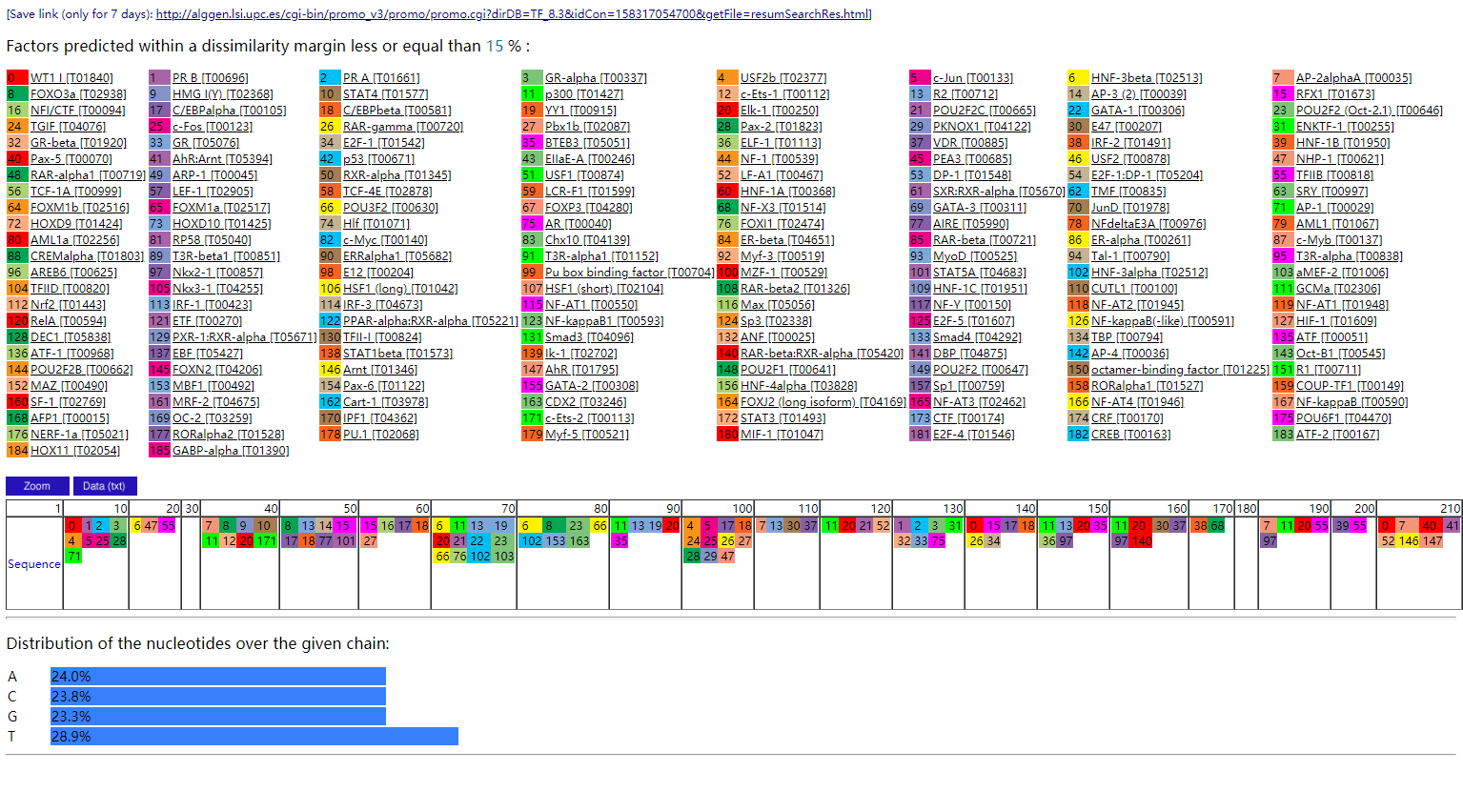


<http://alggen.lsi.upc.es/cgi-bin/promo_v3/promo/promo.cgi?dirDB=TF_8.3&idCon=158317054700&getFile=resumSearchRes.html>

lnc-LRRC1-5

lnc-PRR15L-2


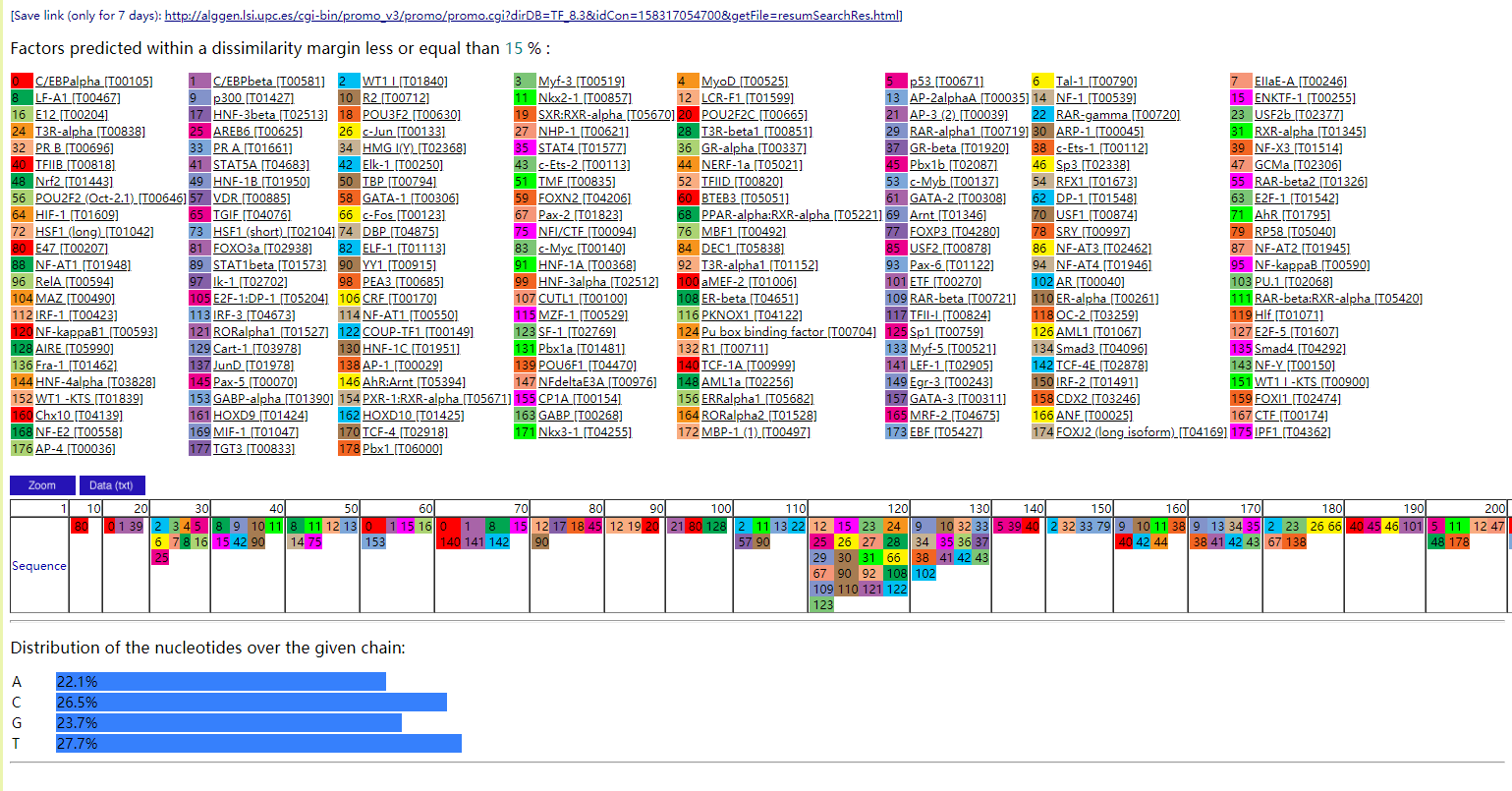


<http://alggen.lsi.upc.es/cgi-bin/promo_v3/promo/promo.cgi?dirDB=TF_8.3&idCon=158317054700&getFile=resumSearchRes.html>

lnc-PRR15L-3


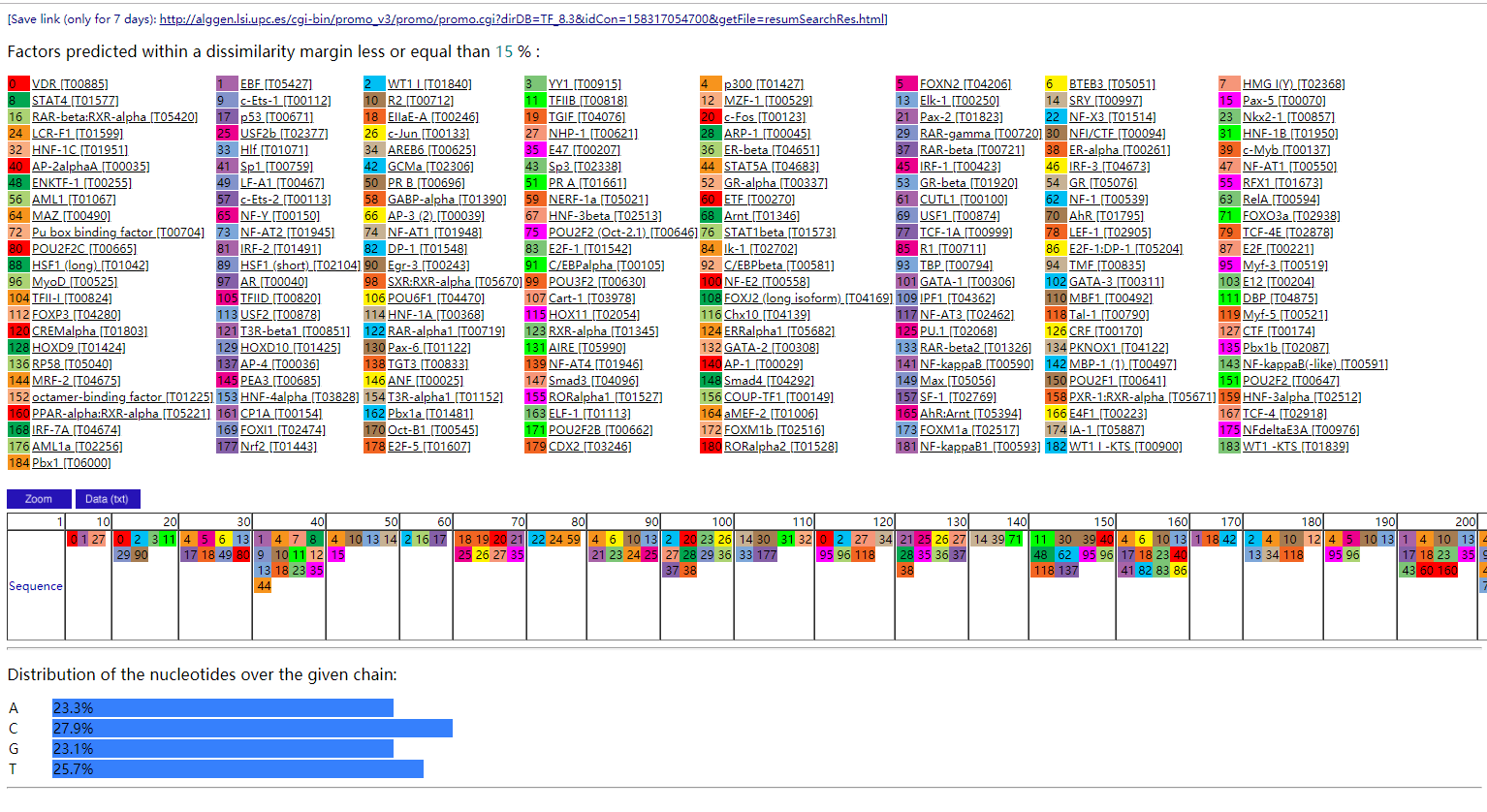


<http://alggen.lsi.upc.es/cgi-bin/promo_v3/promo/promo.cgi?dirDB=TF_8.3&idCon=158317054700&getFile=resumSearchRes.html>

lnc-C4orf21-1

lnc-NT5DC2-1


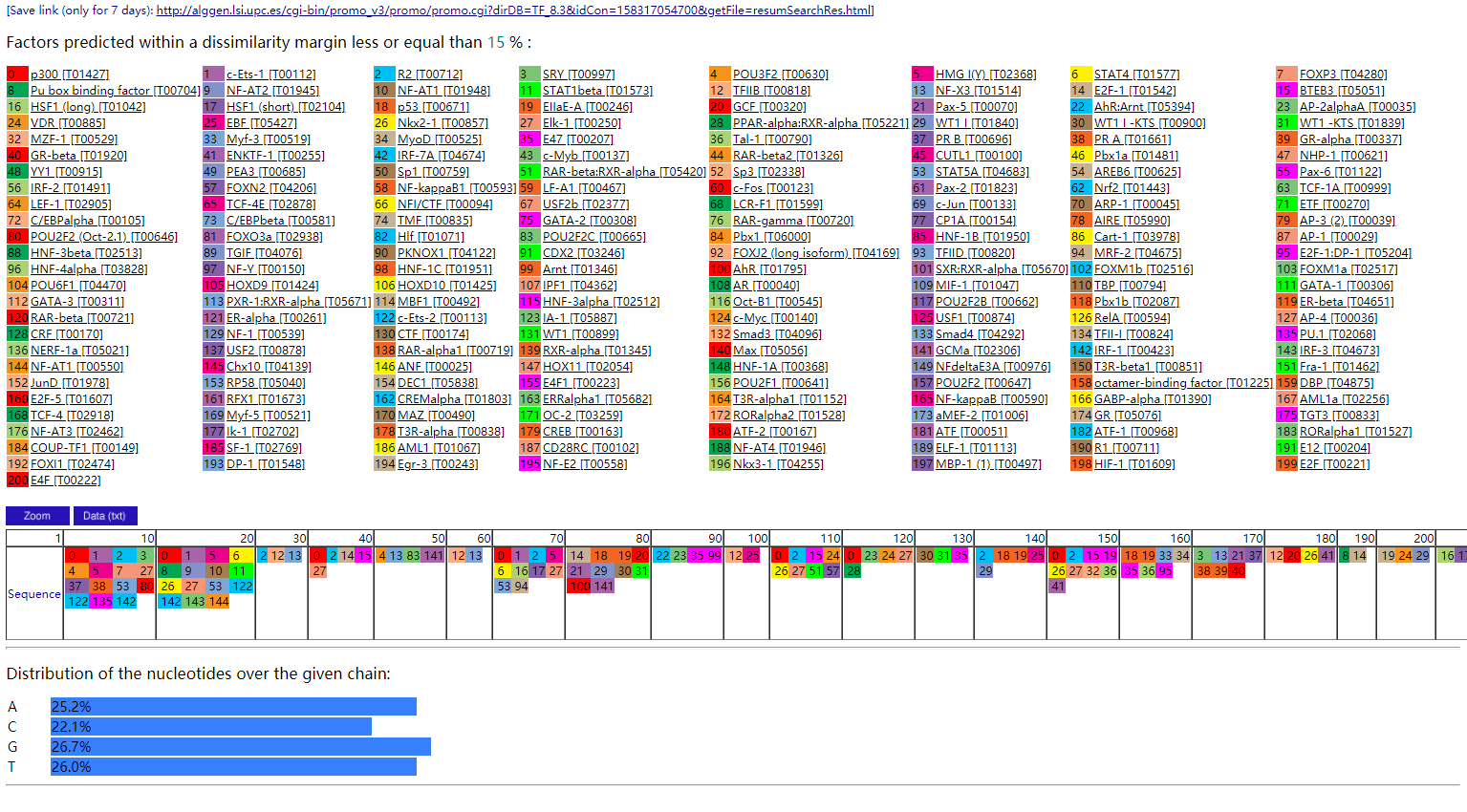


<http://alggen.lsi.upc.es/cgi-bin/promo_v3/promo/promo.cgi?dirDB=TF_8.3&idCon=158317054700&getFile=resumSearchRes.html>

lnc-NUDT5-1


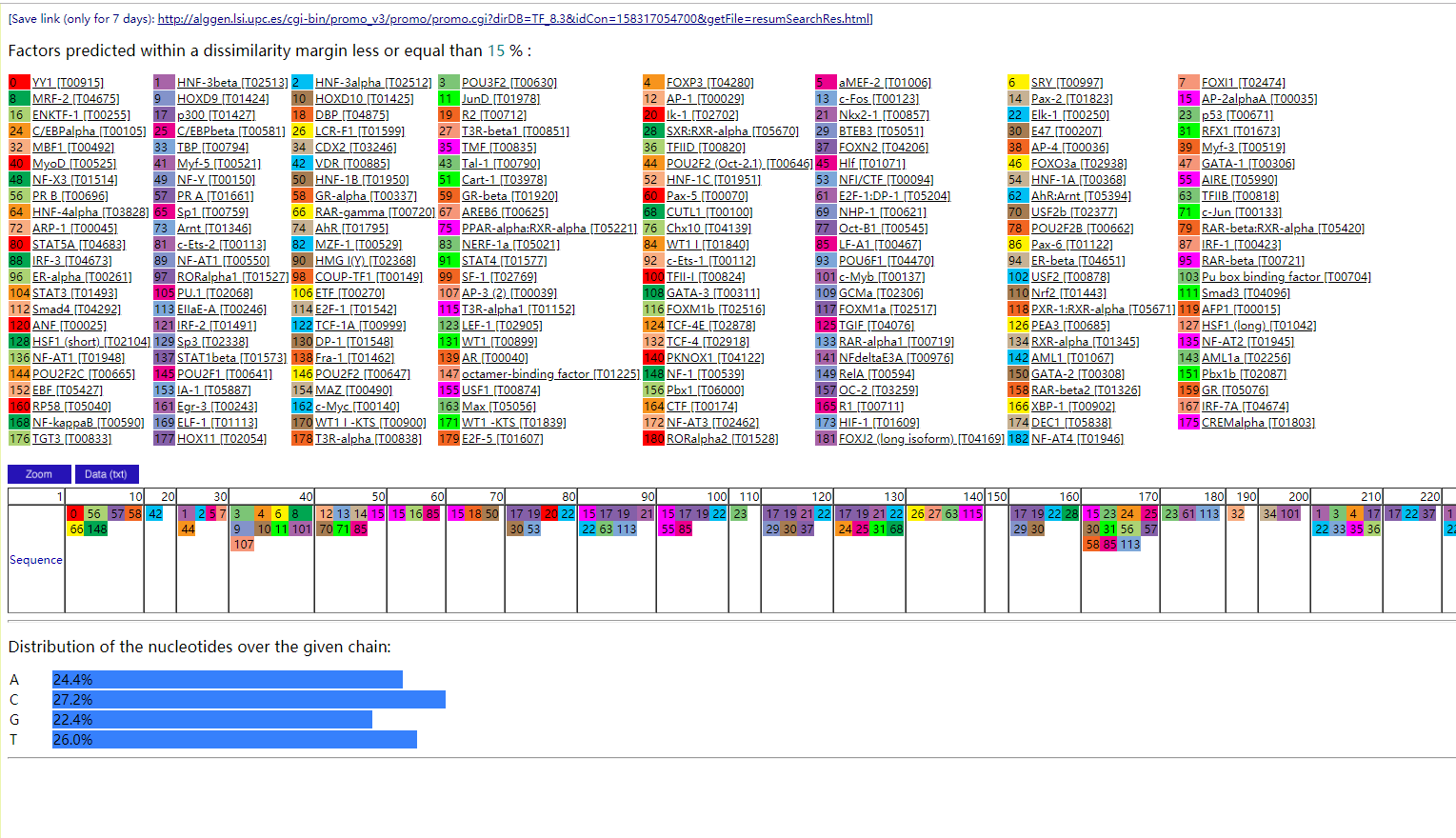


<http://alggen.lsi.upc.es/cgi-bin/promo_v3/promo/promo.cgi?dirDB=TF_8.3&idCon=158317054700&getFile=resumSearchRes.html>

XLOC_000127
