# Supplementary material for: LncRNAs induce oxidative stress and spermatogenesis by regulating endoplasmic reticulum genes and pathways
Source: Aging (Albany NY). 2021 May 6;13(10):13764–87. doi: 10.18632/aging.202971 (PMC8202879; doi:10.18632/aging.202971)
Supplement: Supplementary Table 22 [file aging-13-202971-s022.docx]

**Supplementary Table 22. Associated lncRNAs for crucial genes in endoplasmic reticulum stress, oxidative stress, protein transport and spermatogenesis process.**

| **Biology Process** | **Gene symbol** | **Top strong related lncRNAs (1 >= abs(*r* )> 0.95)** | **Top medium related lncRNAs (0.95 >= abs(*r* )> 0.80)** |
| --- | --- | --- | --- |
| ER Stress | EIF2A | **P:** lnc-FAM198B-1, lnc-C4orf21-1, lnc-SPIN3-5, lnc-AC006050.2.1-6  **N:** | **P:** lnc-TOR3A-1, lnc-STARD9-1, lnc-SLC25A47-5, XLOC_002818  **N:** lnc-TMEM75-8, lnc-CYB5R2-3, XLOC_2993599, lnc-MPP7-8 |
|  | ATF5 | **P:** lnc-ATM-1, lnc-TM2D2-1, XLOC_1484828, lnc-CHAC1-4  **N:** lnc-TMEM75-8 | **P:** lnc-KRT80-10, XLOC_1015300, lnc-PHYHIP-1, lnc-DNASE1L2-1  **N:** lnc-CYB5R2-3, lnc-FMN1-2, MTUS2-AS2, lnc-C1orf106-1 |
|  | ATF4 | **P:**  **N:** | **P:** lnc-KDM4C-18, ARHGEF19-AS1, lnc-FBXL3-1, lnc-LRR1-1  **N:** lnc-C1orf132-1, lnc-CCNYL2-9, lnc-RP3-377D14.1.1-7 |
|  | XBP1 | **P:** lnc-PDF-1  **N:** | **P:** lnc-ABCA5-7, lnc-PHLPP2-1, lnc-AC114947.1.1-1, XLOC_1174715  **N:** lnc-MLLT10-5, lnc-METRNL-1, lnc-SPINT2-5, lnc-KIAA1644-1 |
|  | SERP1 | **P:** lnc-LALBA-1, lnc-FBXL3-1, lnc-C1orf124-1, lnc-MRPS30-8  **N:** | **P:** lnc-CST7-1, lnc-ADC-1, lnc-FLNB-3, lnc-NCKAP1-1  **N:** lnc-C1orf132-1, XLOC_2834725, XLOC_1174819, XLOC_2839990 |
|  | ATP2A1 | **P:** WDFY3-AS2  **N:** | **P:** XLOC_753809, lnc-C1QTNF5-1, lnc-FREM3-6, lnc-SBF2-4  **N:** lnc-BDH1-5, lnc-HSPB1-3, PLCE1-AS2, lnc-RP11-116D17.1.1-4 |
|  | MAPK8 | **P:**  **N:** | **P:**  **N:** lnc-KCNJ13-1, lnc-PHLPP2-1, XLOC_1829856 |
|  | CEBPZ | **P:** lnc-TSPYL4-1, lnc-GPR160-1, XLOC_1484828, lnc-LSM5-2  **N:** lnc-TMEM75-8, lnc-CYB5R2-3 | **P:** lnc-B9D1-1, lnc-TOR3A-1, lnc-ZSCAN21-1, lnc-RNF149-2  **N:** lnc-FMN1-2, MTUS2-AS2, lnc-C1orf106-1, lnc-C20orf187-1 |
|  | BCL2L11 | **P:** XLOC_753555, XLOC_2217616  **N:** | **P:** lnc-SWSAP1-1, ZNF793-AS1, lnc-CCDC70-2, lnc-C2orf40-8  **N:** |
|  | CASP7 | **P:** lnc-C1orf124-1, lnc-REST-4, lnc-FBXL3-1, lnc-MRPS30-8  **N:** | **P:** lnc-LALBA-1, lnc-HAAO-1, lnc-TMEM240-1, XLOC_236009  **N:** lnc-C1orf132-1 |
|  | MCL1 | **P:** XLOC_514590, lnc-PTPN2-6, lnc-GLUD1-2, lnc-TULP4-1  **N:** lnc-C1orf132-1 | **P:** ARHGEF19-AS1, lnc-SPTBN4-3, lnc-NKX3-1-2, lnc-FREM3-2  **N:** XLOC_2837388, XLOC_1174819, XLOC_2834725, XLOC_2396188 |
|  | ERN1 | **P:** XLOC_2836451  **N:** | **P:** lnc-MBTPS2-2, XLOC_2837223, lnc-TAF9-7, lnc-IL17B-5  **N:** lnc-SETDB1-1, XLOC_1176874, lnc-ALX4-7, XLOC_379275 |
|  | EIF2AK1 | **P:** lnc-MPPE1-3, lnc-GYG2P1-1, lnc-AMN-3, lnc-FRMD1-8  **N:** lnc-NMBR-2, lnc-KCNC1-2, lnc-KMO-1, lnc-NR2F2-7 | **P:** lnc-OSBPL7-1, lnc-ITGB3BP-5, H1FX-AS1, lnc-KRT80-9  **N:** lnc-C8orf48-7, lnc-CHRNA5-3, lnc-ROS1-2, lnc-SPIN1-1 |
|  | EIF2AK3 | **P:**  **N:** | **P:** lnc-FAM27B-2, XLOC_1093784, LINC01016, lnc-PRKAB2-2  **N:** lnc-TBCB-1, lnc-FAM214B-2, lnc-GCGR-1, lnc-FARP2-1 |
| Oxidation Stress | SOD1 | **P:** lnc-TULP4-1, lnc-PTPN2-6, FOXP1-AS1, lnc-ZNF101-4  **N:** lnc-C1orf132-1, XLOC_2834725 | **P:** lnc-BCAP31-1, lnc-C2orf84-1, XLOC_2394463, lnc-ADAM21-4  **N:** XLOC_1174819, XLOC_2396188, XLOC_2837388, XLOC_237626 |
|  | SOD2 | **P:** lnc-TCTEX1D1-1, lnc-TMEM240-1, lnc-C1orf124-1, lnc-MRPS30-8  **N:** | **P:** XLOC_753260, lnc-CST7-1, lnc-REST-4, XLOC_1174749  **N:** |
|  | SOD3 | **P:**  **N:** | **P:**  **N:** |
|  | NOX1 | **P:**  **N:** | **P:**  **N:** |
|  | NOX5 | **P:**  **N:** | **P:**  **N:** |
|  | NOXA1 | **P:** lnc-PPIAL4G-5  **N:** | **P:** lnc-ZNF385D-3, lnc-SSTR1-1, LINC01016, lnc-C8orf22-13  **N:** lnc-TBCB-1 |
|  | XDH | **P:** lnc-SEPT7L-2, lnc-POTEF-4, lnc-CLMP-8, lnc-C10orf28-2  **N:** | **P:** lnc-DPY30-4, lnc-TIMM9-3, lnc-GPR157-6, lnc-AC019294.2-1  **N:** lnc-NT5DC2-1, lnc-CHRAC1-3, lnc-TMEM220-1, lnc-TRMT2A-1 |
|  | MAPK14 | **P:** lnc-MKLN1-1, XLOC_1586489, lnc-RBM10-1, lnc-C15orf48-1  **N:** XLOC_379277 | **P:** lnc-AFM-3, XLOC_2032959, lnc-PXDC1-12, lnc-TSHZ3-2  **N:** lnc-ABCD3-1, lnc-RP11-116D17.1.1-4, lnc-ZNF804B-2, LINC00237 |
|  | NFE2L1 | **P:**  **N:** | **P:** MEF2C-AS1, lnc-MTMR2-1, lnc-ERICH1-9, lnc-GAS8-1  **N:** lnc-NDFIP2-13, XLOC_1623391, lnc-RP11-1105G2.3.1-3, lnc-RIPK2-2 |
|  | NFE2L2 | **P:**  **N:** | **P:**  **N:** |
|  | NFKB1 | **P:** lnc-PTEN-12, lnc-NDUFS1-2, lnc-ILK-1  **N:** | **P:** lnc-SCN2A-6, lnc-ZNF732-4, lnc-SWSAP1-1, lnc-CAPN13-1  **N:** XLOC_1547746 |
|  | SP1 | **P:** lnc-CTRB1-2, lnc-KLHL25-12, lnc-HCN3-1, XLOC_515583  **N:** | **P:** lnc-IRF1-4, lnc-PHYHIP-1, lnc-C2orf89-2, lnc-ANKS6-1  **N:** lnc-MPP7-8, XLOC_2839990, XLOC_2993599, lnc-PPP1R3G-3 |
|  | TXN2 | **P:** lnc-ZNF45-2, lnc-PSMC1-1, lnc-PPP1R26-1, PLA2G4C-AS1  **N:** lnc-SUSD1-1, lnc-RP11-324D17.2.1-3, lnc-C20orf187-1, lnc-FMN1-2 | **P:** OXCT1-AS1, XLOC_847185, XLOC_2712257, lnc-FOXN1-1  **N:** XLOC_1830773, lnc-C1orf106-1, lnc-PAH-3, lnc-TMEM75-8 |
|  | GCLC | **P:** lnc-ATM-1, XLOC_1175795, lnc-MFSD2A-1, XLOC_1015300  **N:** | **P:** lnc-AC018816.3.1-9, lnc-LSM5-2, lnc-C3orf24-1, lnc-AC002553.1-1  **N:** XLOC_2993599, lnc-TMEM75-8, XLOC_1547746, lnc-CYB5R2-3 |
|  | HMOX2 | **P:** lnc-TMTC3-14, lnc-FAM164C-2, XLOC_1174283, lnc-DDX19B-2  **N:** | **P:** lnc-ZSCAN21-1, lnc-MRP63-6, lnc-ASPRV1-2, XLOC_1175427  **N:** lnc-AL901608.1-17, XLOC_2835377, lnc-TMEM75-8, lnc-PAH-3 |
|  | HMOX1 | **P:**  **N:** | **P:**  **N:** |
|  | GPX4 | **P:** lnc-REST-4, lnc-GPR37L1-2, lnc-FBXL3-1, lnc-NCKAP1-1  **N:** | **P:** lnc-C1orf124-1, lnc-HAAO-1, XLOC_236009, lnc-LALBA-1  **N:** XLOC_2834725, lnc-C1orf132-1, XLOC_237626 |
|  | GPX1 | **P:**  **N:** | **P:** lnc-RP11-796G6.2.1-3, lnc-CSNK1A1-1, lnc-PSMC3IP-3  **N:** lnc-MORC2-5, lnc-CNOT7-1, lnc-RPL19-6, XLOC_1013595 |
|  | GPX3 | **P:**  **N:** | **P:** lnc-FAM177A1-1, lnc-ATP6V1E2-5, lnc-PDSS1-4, lnc-COL8A2-3  **N:** |
|  | GSR | **P:** lnc-FBXL3-1, lnc-CST7-1, lnc-MRPS30-8, XLOC_1174749  **N:** | **P:** lnc-LALBA-1, lnc-TCTEX1D1-1, lnc-TMEM240-1, lnc-C1orf124-1  **N:** XLOC_2837388, lnc-C1orf132-1 |
|  | NCF1C | **P:** lnc-ZNF804B-1, XLOC_379277, lnc-ABCD3-1, lnc-ZNF804B-2  **N:** lnc-PXDC1-12, lnc-MRPL40-4, lnc-SDCCAG8-1, lnc-AC092295.7.1-2 | **P:** lnc-TBL1XR1-6, lnc-RSPH1-3, lnc-TTC23-5, LINC00237  **N:** lnc-HBG2-1, lnc-PIGG-2, lnc-BTD-1, lnc-AL136219.1-3 |
|  | NCF1 | **P:**  **N:** | **P:**  **N:** |
|  | NCF2 | **P:** lnc-RIOK1-2, XLOC_1236417, lnc-MESDC1-2, lnc-B3GAT1-2  **N:** XLOC_2560875 | **P:** lnc-BNIP2-2, lnc-KDM5B-3, XLOC_2711915, lnc-GPR101-3  **N:** lnc-GANC-1, lnc-STUB1-2, lnc-MRPS25-5, lnc-C16orf62-1 |
|  | NFIX | **P:**  **N:** | **P:** lnc-KIAA1609-1, XLOC_753555, STARD4-AS1, lnc-KDM3A-1  **N:** XLOC_379277, lnc-ABCD3-1, lnc-RP11-116D17.1.1-4, XLOC_2835378 |
|  | FOXM1 | **P:** lnc-RRP1B-1, lnc-BTLA-4, lnc-C18orf62-1, lnc-NRIP1-2  **N:** | **P:** lnc-NQO2-9, XLOC_2712832, lnc-CTA-299D3.8.1-8, lnc-HAUS5-3  **N:** lnc-NDUFAB1-1, lnc-CHAC2-5, lnc-RMI1-1, XLOC_753601 |
| Protein Location | CHMP4B | **P:** lnc-FKBP2-1, lnc-SKA2-2, lnc-FAT2-1, lnc-NDUFAF2-1  **N:** lnc-FMN1-2, lnc-RP11-324D17.2.1-3, lnc-C20orf187-1, lnc-C1orf106-1, | **P:** lnc-SH3RF2-1, lnc-ANKRD27-6, lnc-CSNK1A1-7, lnc-AC091132.1-3  **N:** XLOC_1830773, XLOC_1828024, MEF2C-AS1, lnc-POTEB-5 |
|  | SEC61A1 | **P:** lnc-TCTEX1D1-1, lnc-FLNB-3, lnc-TMEM240-1, lnc-HAAO-1  **N:** | **P:** lnc-CPSF4-1, XLOC_236009, lnc-MRPS30-8, lnc-C1orf124-1  **N:** |
|  | SEC61A2 | **P:** ZNF571-AS1, STARD4-AS1, lnc-AC002553.1-1, lnc-ZNF506-2  **N:** | **P:** XLOC_1484828, lnc-ANKMY2-1, lnc-CCDC148-1, lnc-SETD8-1  **N:** lnc-TMEM75-8, XLOC_2993599, lnc-RP11-116D17.1.1-4, MTUS2-AS2 |
|  | SEC61G | **P:** lnc-TSC2-2, XLOC_2393438  **N:** | **P:** lnc-TRMT61B-1, lnc-MINA-5, lnc-ZNF382-2, lnc-GATM-5  **N:** XLOC_2839036 |
|  | SEC62 | **P:** lnc-C1orf124-1, lnc-TCTEX1D1-1, lnc-TMEM240-1, lnc-CPSF4-1  **N:** | **P:** lnc-MFSD8-1, lnc-REST-4, XLOC_1174749, lnc-CST7-1  **N:** |
|  | SGTA | **P:**  **N:** | **P:**  **N:** |
|  | SGTB | **P:** XLOC_2714543, lnc-C15orf44-1, lnc-RIMBP3C-3, lnc-C19orf44-5  **N:** | **P:** lnc-LYRM2-1, lnc-PIGN-1, lnc-RPS6-3, lnc-PPIC-6  **N:** |
|  | SPCS1 | **P:** lnc-MTRR-7, lnc-AMDHD2-2, lnc-KCNT1-7, lnc-H3F3B-1  **N:** | **P:** lnc-DSCC1-1, lnc-MYL6-2, lnc-HMGB2-15, lnc-HCRTR1-1  **N:** lnc-PADI1-1, lnc-ROS1-2, lnc-SYCP1-1, XLOC_1483319 |
|  | SPCS2 | **P:**  **N:** | **P:**  **N:** |
|  | SPCS3 | **P:** lnc-PTRHD1-1, lnc-LAMA5-3, lnc-RAB15-1, lnc-HEATR4-6  **N:** | **P:** lnc-FAM117A-1, lnc-FAM8A1-2, XLOC_1013783, lnc-GYG2P1-1  **N:** XLOC_1483319, lnc-OAZ3-4, lnc-SYCP1-1, lnc-FAM120B-3 |
|  | SRP9 | **P:** lnc-IARS2-2, lnc-WDR77-2, lnc-C1QL3-1, lnc-SLC36A1-5  **N:** | **P:** lnc-RAD9B-1, lnc-ZNF687-1, lnc-SLC3A2-3, lnc-MAP6-1  **N:** lnc-PPP1R3G-3, lnc-GGCT-1, XLOC_2028132, lnc-MPP7-8 |
|  | SRP14 | **P:**  **N:** | **P:** lnc-BCAP31-1, XLOC_1829151, ARHGEF19-AS1, XLOC_2217694  **N:** lnc-C1orf132-1, MEG8 |
|  | SRP19 | **P:** lnc-TCTEX1D1-1, lnc-HAAO-1, lnc-CPSF4-1, lnc-TMEM240-1  **N:** | **P:** OIP5-AS1, XLOC_236009, lnc-ADC-1, lnc-NCKAP1-1  **N:** |
|  | SRP72 | **P:**  **N:** | **P:** lnc-RBM10-1, XLOC_2032959, lnc-C6orf146-4, XLOC_1586489  **N:** XLOC_379277, lnc-CGNL1-3, lnc-RP11-116D17.1.1-4, XLOC_2218688 |
|  | SRP54 | **P:**  **N:** | **P:**  **N:** |
|  | SSR3 | **P:** lnc-MRPS30-8  **N:** | **P:** OIP5-AS1, lnc-TMEM240-1, XLOC_236009, lnc-C1orf124-1  **N:** lnc-C1orf132-1 |
|  | TRAM1 | **P:**  **N:** | **P:** lnc-HAAO-1, lnc-LALBA-1, lnc-NCKAP1-1, XLOC_2217694  **N:** |
|  | TRAM2 | **P:**  **N:** | **P:** lnc-KHDRBS3-4, lnc-DNAJA1-2, lnc-C20orf20-1, lnc-C15orf41-11  **N:** |
|  | RYR2 | **P:** lnc-RP3-377D14.1.1-7  **N:** lnc-DDHD2-1, XLOC_845984 | **P:** lnc-MBTPS2-1, lnc-FBXO11-1, lnc-S1PR1-2, lnc-GPR157-6  **N:** XLOC_2032021, lnc-NT5DC2-1, MRVI1-AS1, XLOC_2031508 |
|  | ANK2 | **P:** MEF2C-AS1, lnc-C20orf187-1, lnc-CYB5R2-3, lnc-PAH-3  **N:** lnc-FOXN1-1, PITPNA-AS1, lnc-CTSZ-7, lnc-MORC2-1 | **P:** lnc-TMEM75-8, XLOC_1828024, lnc-ATP6V1G3-4, ITGA9-AS1  **N:** lnc-SKA2-2, lnc-ADAMTS18-1, TAB3-AS2, lnc-TUSC5-5 |
|  | DDRGK1 | **P:**  **N:** | **P:**  **N:** |
|  | INSIG1 | **P:**  **N:** | **P:** lnc-AC092295.7.1-2, lnc-PIGG-2, lnc-SLC36A1-5, lnc-C1QL3-1  **N:** XLOC_845107, XLOC_2028132, MTUS2-AS2, lnc-MPP7-8 |
|  | KDELR2 | **P:**  **N:** | **P:** lnc-EDC3-3, lnc-PANK3-13, OIP5-AS1, lnc-C11orf1-1  **N:** |
|  | RER1 | **P:** lnc-GDPD4-1, lnc-RP1-286D6.2.1-2, lnc-NMNAT1-3, lnc-RRP8-1  **N:** | **P:** lnc-WNT8A-2, lnc-PTGDR2-2, lnc-C1orf122-2, lnc-MYOM1-1  **N:** lnc-TFAP2C-4, XLOC_1621025, lnc-SP3-1, XLOC_1229860 |
|  | RTN4 | **P:**  **N:** | **P:** lnc-LALBA-1, lnc-TCTEX1D1-1, lnc-TMEM240-1, lnc-CPSF4-1  **N:** |
|  | TAPT1 | **P:** lnc-ACMSD-6, lnc-DPY30-4  **N:** | **P:** lnc-C8orf83-1, lnc-ADAMTS16-2, lnc-NTF3-3, lnc-TRMT11-1  **N:** lnc-CHRAC1-3, RNF185-AS1, XLOC_2396781, lnc-C7orf23-2 |
|  | UBAC2 | **P:**  **N:** | **P:** lnc-CEBPG-4, lnc-F8-1, lnc-IMPDH2-2, XLOC_004093  **N:** lnc-RCC1-6, lnc-GUCY1A3-3, lnc-SP3-1, XLOC_1621025 |
| Spermatogenesis | HOXA11 | **P:** lnc-RP11-404P21.6.1-9, lnc-ENTPD5-5, LINC00894, lnc-C15orf32-3  **N:** | **P:** lnc-C17orf97-5, lnc-SLC30A4-2, lnc-BPHL-9, lnc-LMOD1-4  **N:** lnc-GUCY1A3-3, XLOC_2217124, lnc-MYC-3, lnc-RCC1-6 |
|  | HOXD9 | **P:**  **N:** | **P:** lnc-CDK20-4, XLOC_2218688, lnc-GALNTL2-1, lnc-CGNL1-3  **N:** lnc-TNNC2-1, lnc-RP11-279O9.4.1-1, lnc-C5orf39-1, lnc-MAFF-6 |
|  | LHCGR | **P:** lnc-DPY30-4, lnc-ACMSD-6, lnc-ADAMTS16-2  **N:** | **P:** lnc-RPS24-3, lnc-NTF3-3, lnc-AC073416.2-11, lnc-DOC2B-3  **N:** XLOC_2396781, RNF185-AS1, lnc-LRRC3C-2, lnc-COX7A2L-4 |
|  | MAP3K1 | **P:**  **N:** | **P:** TRAF3IP2-AS1, LINC00842, lnc-CLVS1-1, lnc-LEKR1-1  **N:** XLOC_2394367, lnc-IL10RB-3, XLOC_2396935, lnc-WDR83OS-1 |
|  | SOX12 | **P:**  **N:** | **P:**  **N:** XLOC_515394, lnc-DNAJC5B-1, lnc-EDC3-3, lnc-DENND4C-1 |
|  | SOX5 | **P:** XLOC_1093784, lnc-NTF3-3, lnc-DPY30-4, lnc-ADAMTS16-2  **N:** | **P:** lnc-C8orf83-1, lnc-ACMSD-6, lnc-DOC2B-3, lnc-TRMT11-1  **N:** lnc-CHRAC1-3, RNF185-AS1, lnc-ERH-1, lnc-SLC7A7-2 |
|  | SOX17 | **P:**  **N:** | **P:** lnc-CHD2-3, lnc-ATP1A3-1, lnc-F13A1-3, lnc-HSPA12B-1  **N:** LINC00689, lnc-IL6-3 |
|  | SPATA21 | **P:**  **N:** lnc-MARCH10-1, lnc-ZNF146-1, lnc-KCNT1-7, lnc-PPP4C-8 | **P:** lnc-RCC1-6, XLOC_2839990, XLOC_2993599, lnc-PADI1-1  **N:** lnc-AC127496.3-6, lnc-HCRTR1-1, lnc-TOP1MT-5, lnc-C1QTNF5-1 |
|  | SPATS2 | **P:** lnc-AL033381.1-4, lnc-SPP1-1, lnc-APEX1-2, lnc-RP11-404L6.2.1-4  **N:** lnc-LEP-1, XLOC_379275, lnc-USP47-2, lnc-RD3-5 | **P:** lnc-IGFN1-5, XLOC_2217825, lnc-EXOC2-21, lnc-NCEH1-1  **N:** XLOC_001052, lnc-SPG11-3, lnc-RPS4XP21-1, lnc-C4orf37-2 |
|  | SOHLH1 | **P:** lnc-BACE1-2  **N:** | **P:** lnc-CAP1-1, lnc-SAMD5-1, LINC01359, lnc-RP11-180C1.1.1-8  **N:** lnc-HOXA13-4, lnc-NFATC3-1, lnc-TMED5-5, BIRC6-AS1 |
|  | ADCYAP1R1 | **P:** lnc-RRP1B-1, lnc-NRIP1-2  **N:** | **P:** XLOC_002503, lnc-C18orf62-1, XLOC_2712832, lnc-C15orf41-11  **N:** lnc-NDUFAB1-1, lnc-CHAC2-5, lnc-RMI1-1, XLOC_379275 |
|  | TYRO3 | **P:** lnc-IL17RA-13, SATB1-AS1, lnc-ZNF30-1, lnc-BOD1-1  **N:** | **P:** lnc-AC107021.1-9, lnc-AC073416.2-11, lnc-RPS24-3, lnc-NCEH1-1  **N:** lnc-LEP-1, lnc-RD3-5, XLOC_001052, lnc-USP47-2 |
|  | AXL | **P:**  **N:** | **P:** XLOC_2557803  **N:** |
|  | SPAM1 | **P:** lnc-PPIAL4G-5, lnc-C8orf22-13, LINC01016  **N:** lnc-TBCB-1 | **P:** lnc-ZNF385D-3, lnc-FAM27B-2, lnc-CBWD5-2, lnc-RPL19-6  **N:** lnc-NDUFAB1-1, lnc-C7orf23-2, lnc-MATN2-1, lnc-CCDC51-1 |
|  | MTA3 | **P:**  **N:** lnc-OXSM-2, lnc-ZNF775-1, lnc-RP11-1105G2.3.1-3 | **P:** lnc-CYB5R2-3, lnc-C20orf187-1, lnc-ERICH1-9, MEF2C-AS1  **N:** XLOC_1623391, lnc-NDFIP2-13, lnc-POC1B-GALNT4-2, lnc-RIPK2-2 |
